# Analysis of spatial-temporal gene expression patterns reveals dynamics and regionalization in developing mouse brain

**DOI:** 10.1038/srep19274

**Published:** 2016-01-20

**Authors:** Shen-Ju Chou, Chindi Wang, Nardnisa Sintupisut, Zhen-Xian Niou, Chih-Hsu Lin, Ker-Chau Li, Chen-Hsiang Yeang

**Affiliations:** 1 Institute of Statistical Science, Academia Sinica, Nankang, Taipei, Taiwan; 2 Institute of Cellular and Organismic Biology, Academia Sinica, Nankang, Taipei, Taiwan

## Abstract

Allen Brain Atlas (ABA) provides a valuable resource of spatial/temporal gene expressions in mammalian brains. Despite rich information extracted from this database, current analyses suffer from several limitations. First, most studies are either gene-centric or region-centric, thus are inadequate to capture the superposition of multiple spatial-temporal patterns. Second, standard tools of expression analysis such as matrix factorization can capture those patterns but do not explicitly incorporate spatial dependency. To overcome those limitations, we proposed a computational method to detect recurrent patterns in the spatial-temporal gene expression data of developing mouse brains. We demonstrated that regional distinction in brain development could be revealed by localized gene expression patterns. The patterns expressed in the forebrain, medullary and pontomedullary, and basal ganglia are enriched with genes involved in forebrain development, locomotory behavior, and dopamine metabolism respectively. In addition, the timing of global gene expression patterns reflects the general trends of molecular events in mouse brain development. Furthermore, we validated functional implications of the inferred patterns by showing genes sharing similar spatial-temporal expression patterns with *Lhx2* exhibited differential expression in the embryonic forebrains of *Lhx2* mutant mice. These analysis outcomes confirm the utility of recurrent expression patterns in studying brain development.

Neural development is a highly complex process unfolding in space and time. During the course of development, the neural plate is transformed into a convoluted brain shape with many specialized regions; neuroectoderm stem cells are differentiated into hundreds of cell types; billions of neural cells migrate to specified locations and form an astronomical number of connections^1^. Different cell types in distinct brain regions and developmental stages are engaged in different functions, which are accomplished by different sets of genes. Therefore, transcription profiles in developing brains are highly heterogeneous (in terms of locations), dynamic (in terms of time), and diverse (in terms of genes).

Advanced imaging and genomic technologies enable neural biologists to map the connections, functions and gene expression profiles of brain regions. There are already several “atlases” of human and mouse brains generated by the Allen Brain Institute, providing comprehensive expression profiles of thousands of genes in refined brain structures and connections of defined regions and cell types. They include the databases of adult mouse brain gene expressions^2^, developing mouse brain gene expressions^3^, prenatal human brain gene expressions^4^, adult human brain gene expressions^5^, and a mesoscale connectome of mouse brains^6^. Among them the developing mouse brain database of the Allen Brain Atlas comprises unique spatial-temporal-gene expression data. It probes only about 2100 genes but covers their expression profiles in the anatomical structures of the whole brain at seven developmental time points.

The complexity in space, time and genes poses a great challenge in extracting useful information from this dataset. Currently, most studies utilize the brain atlas data with three approaches: (1) demarcating the expressed regions of selected genes^7^,^8^,^9^,^10^,^11^,^12^,^13^, (2) fishing out the genes expressed in selected regions and/or time points^14^,^15^,^16^,^17^,^18^,^19^,^20^, (3) comparing the expression profiles of multiple regions or genes^21^,^22^,^23^,^24^,^25^,^26^,^27^,^28^,^29^. Despite the rich knowledge derived from each approach, their computational methods did not explicitly incorporate the structures underneath the spatial-temporal data. Instead, spatial-temporal properties emerge from the analysis outcomes. For instance, regions sharing similar expression profiles tend to be connected^27^ or share the same cell types or anatomical structures^25^; inter-regional divergence of expression profiles is high in early embryos and adults but reaches a low point around birth^26^.

Several lines of computational research built more structured models of the brain expression data beyond pattern match and correlations. Most of them applied standard tools of gene expression data analysis such as matrix factorization^30^,^31^,^32^ and regression models^33^. These studies assumed the expression data is a collection of independent instances sampled from regions or voxels of the brain images, thus dropped the information about spatial dependency of sampled regions/voxels. A few other studies incorporated spatial information for comparing brain expression images^34^ and mapping the 3D gene expression data onto a flat chart^35^. These studies tackled primarily the spatial dimension since their data usually did not include the temporal aspect. Many studies of gene expression data analysis tackled the dynamic nature of gene regulation events^36^,^37^,^38^, yet the spatial aspects were often not addressed as spatial information was missing in most gene expression datasets.

Beyond brain development, spatiotemporal gene expression analysis is commonly performed in developmental biology^39^,^40^. Single-cell sequencing technologies enable biologists to track the gene expression dynamics across multiple developmental lineages^41^. However, the majority of analysis approaches still fall into the three aforementioned categories and replace spatial information with cell types or developmental lineages. In contrast, sophisticated quantitative tools tackling spatiotemporal patterns are proposed by mathematical biologists. Pattern formation is a highly developed sub-discipline dated back to Turing’s seminal paper of reaction-diffusion models^42^. Since then many sophisticated models and simulation techniques have been developed^43^,^44^. Yet those studies primarily address modeling and simulation aspects rather than the data analysis aspect.

In this study, we proposed a novel method to reduce the complex brain gene expression data into superposition of recurrent patterns, while preserving the spatial information in the patterns and comparing the temporal variations of those patterns. It consists of the following key reduction procedures. First, we projected the expression of each gene on a 2D grid of anatomical substructures. Second, we demarcated the expressed regions on the 2D grid. Third, we clustered the expressed regions of all genes and all time points to obtain the recurrent patterns either in the entire brain or in localized areas. The spatial-temporal patterns inferred from the Allen Developing Mouse Brain Atlas conformed with knowledge of mouse brain development and were validated by additional experiments. The timing of global gene expressions reflects the general trends of molecular events in mouse brain development. Localized gene expression patterns reveal regional distinction in mouse brain development. Moreover, we verified the functional implication of inferred spatial-temporal patterns by measuring the transcriptomic responses of the cortical-specific deletion of *Lhx2*, a critical transcription factor for cortical development. The analysis outcomes demonstrate the utility of recurrent patterns to comprehend the complex spatial-temporal brain expression data.

## Results

### Delineating spatial-temporal patterns of mouse brain gene expressions

The Allen Brain Atlas (ABA^45^) provides by far the most comprehensive data of gene expressions in human and mouse brains. The developing mouse brain atlas of ABA consists of the *in-situ* hybridization experimental data of about 2100 genes, 434946 images, and 7 developmental stages – E11.5, E13.5, E15.5, E18.5, P4, P14, and P28^3^. ABA categorizes brain substructures into 10 ontological levels that basically reflect a simplified sequence of regionalization events in mouse brain development. Higher-level substructures possess finer-grained characteristics and are emerged at later developmental stages. We focused on level 5 substructures as they comprised all the segments along the anterior-posterior axis and all the four longitudinal zones along the dorsal-ventral axis (Supplementary Figure S1 and the Allen Brain Atlas website, http://developingmouse.brain-map.org/).

**Figure 1:**
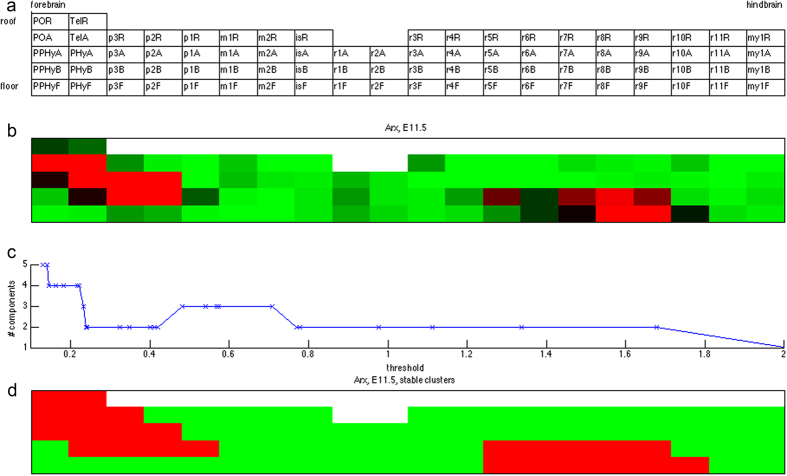
Illustration of quantization for ABA gene expression data. (**a**) A rectangular grid derived from the level 5 substructures. Left: forebrain (anterior), right: hindbrain (posterior), top: roof (dorsal), bottom: floor (ventral). (**b**) The spatial expression profile of Arx at E11.5. Red and green blocks denote substructures with high and low expression levels respectively. White blocks denote either non-existing substructures or missing data. (**c**) The number of expressed regions obtained from the quantized Arx expression data with varying threshold values. (**d**) The quantized Arx expression data with threshold 0.2384.

The topology of level 5 substructures can be deformed into a rectangular grid with a slight discrepancy (Fig. 1a). The grid consists of 20 segments along the anterior-posterior axis, 4 layers along the dorsal-ventral axis, and totally 80 blocks (substructures). Discrepancy occurs at only two interfaces: between TelA (alar plate of evaginated telencephalic vesicle) and p3R (prosomere 3 roof plate), and between r1A (rhombomere 1 alar plate) and isR (isthmic roof plate). Conforming with the grid topology, the gene expression data is represented as a vector field 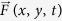, where 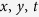 are coordinates along the anterior-posterior, dorsal-ventral and temporal axes. Each component 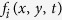 of 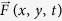 corresponds to the expression levels of gene 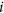 over space and time.


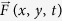
 is a high-dimensional tensor data possessing complicated structures. To extract information about brain development, one has to reduce and decompose 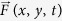 into simplified and tractable forms. In this study, we provided three simplification procedures. First, for each gene and each time point, we identified contiguous regions where the expression levels were elevated. Second, for each gene we constructed a binary vector of global expression states over seven time points, indicating whether it is expressed in a large portion of the brain at each time point. We then grouped the genes sharing the same global expression states together. Third, by assembling the expressed regions with overlapped substructures, we inferred the “local patterns” that recurred in multiple genes and time points. The expression profile of each gene is the superposition of a global expression state and the local patterns. Procedures of demarcating expressed regions, generating global expression states and local patterns and their validation are described in Materials and Methods.

### The timing of global gene expressions reflects the general trends of molecular events in mouse brain development

For each gene and time point, we demarcated the expressed regions by applying a binary quantization to the data. The robust clusters over a wide range of threshold values were extracted. The procedures of expressed region demarcation are illustrated in Fig. 1b–d and described in Materials and Methods.

We labeled a gene as globally expressed (as one) at a time point if the number of expressed substructures in the grid exceeded half of the total number of substructures with observed data, otherwise, as zero. Accordingly, each gene gives rise to a binary global expression state vector over seven time points. Table 1 summarizes the number of genes possessing each global expression state vector. Intriguingly, the all-zeros and all-ones states cover the highest numbers of genes (453 and 235 respectively). Non-globally expressed states (zero entries in the binary global expression state vectors) include two possible cases: existence of locally expressed patches and no expressions. The former cases are more frequent than the latter, as the average number of expressed substructures among the 7623 gene-time point combinations with the zero state is 9.01. Therefore, abundance of genes with all-zeros states implies many genes exhibit localized expressions over all developmental stages. Conversely, a large number of genes also exhibit global expressions over all time points.

Most of the remaining top-ranking global expression states tend to have zeros in the first few time points and ones in the postnatal stages. Among the top 10 global expression states following all zeros and all ones (ranks 3-12 in Table 1), 10, 9 and 8 of them have zeros at E11.5, E13.5 and E15.5 respectively, and 8 and 7 have ones at P14 and P4 respectively.

**Figure 2:**
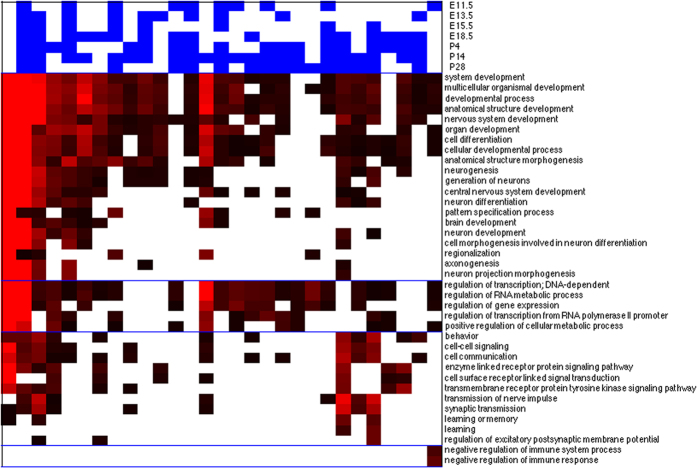
Functional enrichments of global expression states. The enrichment outcome of each global expression state is displayed in one column. The top portion of a column denotes the binary global expression states over 7 time points (from E11.5 to P28). Blue and white patches indicate 1s and 0s. The remaining portion of the column denotes the enrichment p-values in selected GO categories. Each row denotes a GO category. Bright red patches indicate high 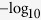
p-values, black patches indicate low 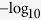 p-values, and white patches indicate enrichment p-values are not available. The GO categories are divided into four classes: developmental genes, transcription factors, neural information processing and immune response genes. The GO categories in these classes are separated by blue lines.

We then assessed the Gene Ontology (GO) functional enrichment of genes possessing each global expression state. The genes appeared in the ABA data were highly biased toward functions pertaining to brain development and/or neural information processing by selection^3^. We divided the enriched GO categories into four broad classes: (1) brain development, (2) transcription factors, (3) neural information processing, and (4) immune responses. Figure 2 displays the hyper-geometric enrichment p-values of global expression states in selected GO categories of these classes. The genes belonging to each global expression state and complete enrichment outcomes of GO categories are reported in Supplementary Datasets S1 and S2 respectively.

Global gene expression states enriched with genes in each functional class possess distinct characteristics. The top two global expression states (all zeros and all ones) have highly significant enrichment p-values in developmental genes and transcription factors. This is expected since the two global expression states contain many genes and these two functional classes are highly representative among the probed genes. In contrast, neural information processing genes tend to have non-globally expressed states (0 entries) at early stages and globally expressed states (1 entries) at the remaining time points. For instance, the three most enriched states (0001111, 0011111, 0111111) for neural information processing genes (“behavior” – GO:0007610 and “synaptic transmission” – GO:0007268) depict three common spatiotemporal expression patterns – unexpressed or only expressed in part of the brain up to a certain time point (E15.5, E13.5, E11.5 respectively) at early stages and then become globally expressed afterwards (after time point E18.5, E15.5, E13.5 respectively) (see Supplementary Dataset S2 for enrichment p-values).

Besides the all-zeros and all-ones vectors, transcription factors tend to have globally expressed states at postnatal stages and non-globally expressed states at the remaining time points. For instance, the most enriched global expression states for “regulation of RNA metabolic process” (GO:0051252) are 0000000, 1111111, 0000010, and 0000001 (see Supplementary Dataset S2 for enrichment p-values).

Brain developmental genes seem to possess several global expression states. Beyond the “usual suspects” of all-zeros and all-ones vectors, the most enriched global expression states for general system development (e.g., “system development”, GO:0048731, “multicellular organismal process”, GO:0032501) are 0111111 and 0000010. However, the genes involved in neural system development (e.g., “nervous system development”, GO:0007399, “neurogenesis”, GO:0022008) are enriched with 0111111 alone. Curiously, only one global expression state 1000000 is enriched with immune response genes (e.g., negative regulation of immune response, GO:0050777).

### Localized gene expression patterns reveal regional distinction in mouse brain development

The global expression states indicate the prevalent presence of genes with general-purpose functions but do not suffice to illuminate regional distinction in brain development. Beyond the global expressions, multiple genes are co-expressed at specific regions at multiple time points. We define a recurrent local expression pattern as a collection of overlapping and contiguous areas which are demarcated as expressed regions in multiple gene-time point combinations. To recapitulate these patterns, we extracted the gene-time point combinations not labeled as global expression states, demarcated their contiguous expressed regions, then applied a graph theory based clustering algorithm to group those contiguous regions. The algorithm is described in Materials and Methods and Supplementary Information.

An example of the recurrent local spatial expression pattern is illustrated in Supplementary Figure S2 (pattern 45). It is shared by 10 gene-time point combinations in 10 genes. Each gene contains a small patch of expressed region in the basal/alar layers of the middle of hindbrain.

**Figure 3:**
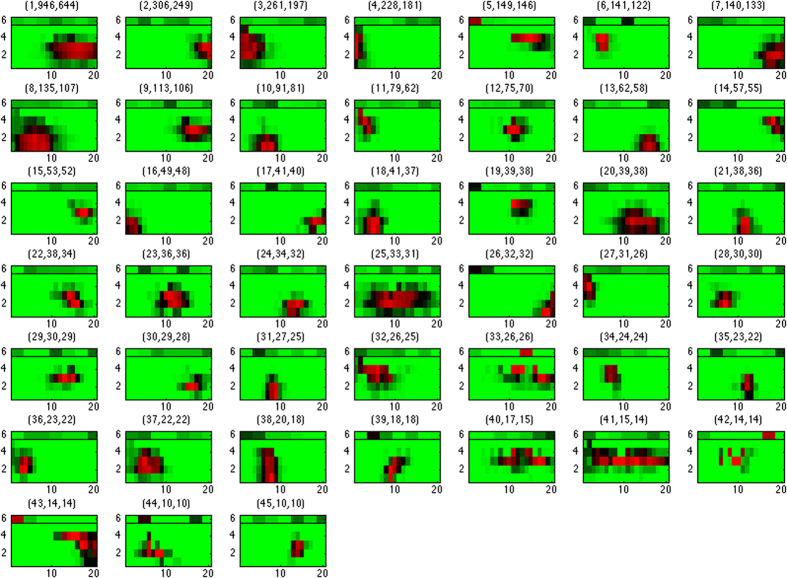
45 recurrent local spatial expression patterns. In each panel, the three numbers denote the index of the pattern, numbers of its occurrences among all (gene,time) combinations and among all genes. The top horizontal bar visualizes the relative frequencies of the pattern’s occurrence at 7 time points. Bright red pacthes and bright green patches represent the highest and lowest relative frequencies respectively. The spatial location and distribution of a pattern is visualized on a grid. The color in each substructure block represents the relative frequency where it is activated among the constituting expressed regions of a pattern. The color code follows the horizontal bars. The orientation of the grids conforms with Fig. 1a.

Figure 3 summarizes the 45 recurrent local expression patterns derived from the clustering algorithm. They are sorted by the numbers of occurrences among the gene-time point combinations (the second numbers in the three-component vectors on top of each pattern) and among the genes (the third numbers in the vectors). Since each pattern is a collection of overlapping regions, we represent it as a distribution over substructures on the 2D grid: the color of grids denote the frequency where each substructure occurs in the collection of overlapping regions.

These patterns exhibit diverse spatial and temporal variations. Some top-ranking patterns span wide spatial ranges. For instance, pattern 1 covers the alar and basal layers along the entire hindbrain, and pattern 8 covers the ventral regions in the forebrain. Others occupy narrow regions. For instance, pattern 4 covers the prosencephalon and pattern 6 covers the alar and basal layers of prosomeres 1 and 2. Moreover, some patterns occur at only specific time points. For instance, pattern 5 occurs primarily at E11.5, and pattern 6 occurs at only E11.5 and E18.5, while others can occur at all time points, though on different genes (e.g., patterns 1 and 4).

**Figure 4:**
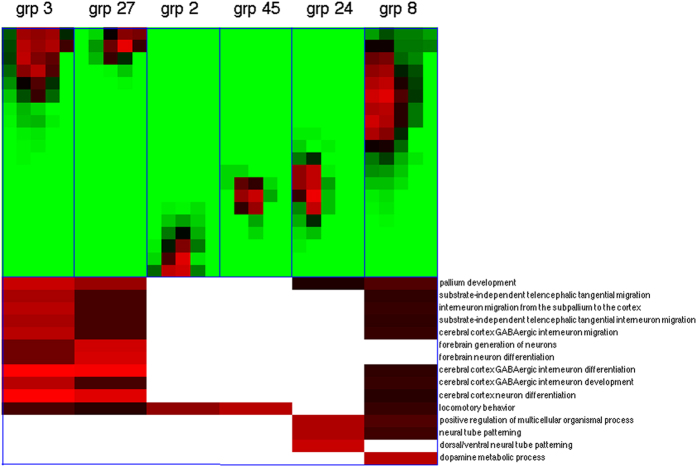
Functional enrichments of 6 spatial expression patterns in selected GO categories. The spatial location and distribution of each pattern is again marked on a grid with an orientation rotated 90 degrees clockwise: top: anterior, bottom: posterior, right: dorsal, left: ventral. The color code of the pattern locations/distributions follows Fig. 3. The color code of enrichment p-values follows Fig. 2.

Analogous to global expression states, we also examined the functional enrichments of genes possessing each spatial expression pattern. Strikingly, we found that the locations of some spatial patterns match the targeted brain regions of their enriched GO categories. Figure 4 displays the enrichment p-values of selected patterns and GO categories. Patterns 3 and 27 are active in the forebrain regions and enriched with several functional categories involved in forebrain development, such as pallium development (GO:0021543), generation of neurons in the forebrain (GO:0021872), and cerebral cortex GABAergic interneuron differentiation (GO:0021892). In contrast, patterns 2 and 45 are activated in the posterior end (medullary) and middle (pontomedullary) of the hindbrain and enriched with the GO category of locomotory behavior (GO:0007626). These regions are close to cerebellum, which is the motor control center of the brain. Moreover, pattern 8 is active in the floor and basal layers of forebrain and enriched with dopamine metabolic process (GO:0042417). The activated region in pattern 8 coincides with basal ganglia and dopamine plays an important role in its function^46^. The genes possessing each local pattern and the complete GO category enrichment outcomes of spatial patterns are reported in Supplementary Datasets S3 and S4 respectively.

### Expressions of most genes are not reduced to single spatial patterns

**Figure 5:**
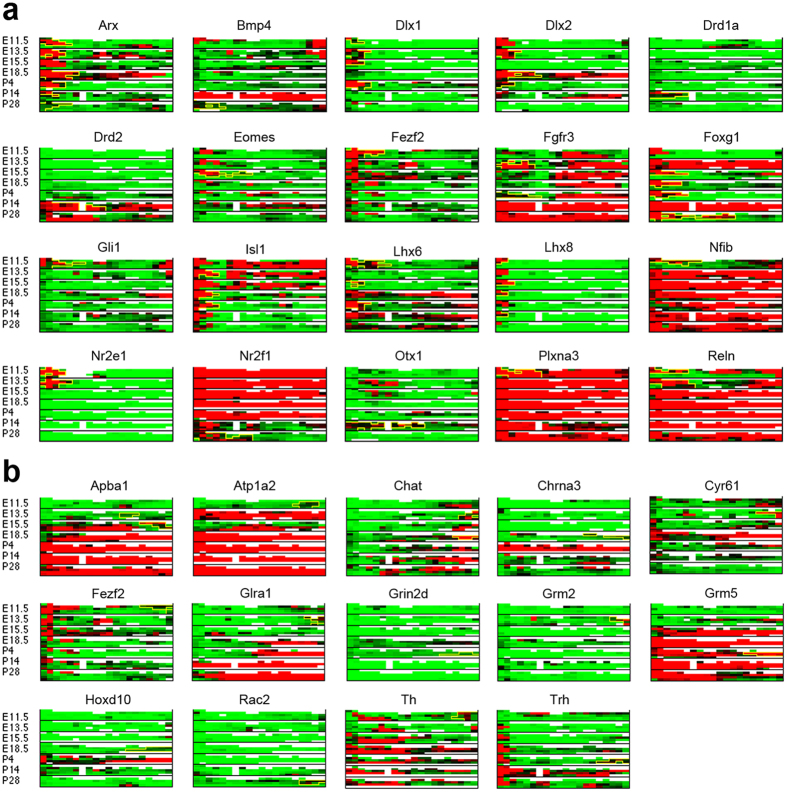
The spatial-temporal gene expression profiles show correlations with area-specific functions. (**a**) The spatial-temporal expression profiles of genes possessing pattern 3 or 27 and involved in forebrain development. The spatial expression profiles at 7 time points are stacked vertically. The grid orientation and color code of expression levels follow Fig. 3. The boundaries of expressed regions belonging to pattern 3 or 27 in each gene are marked by yellow lines. (**b**) The spatial-temporal expression profiles of genes possessing patterns 2 or 45 and involved in locomotory behavior.

Global expression states and spatial patterns constitute the building blocks of spatial-temporal expression profiles of genes. Most gene expression profiles are decomposed as a superposition of multiple building blocks. To demonstrate the non-local nature of gene expressions, we visualize the expression data of two groups of genes in Fig. 5. Group 1 constitutes the intersection of genes possessing pattern 3 or 27 (forebrain-activated regions) and genes in the 10 forebrain-specific development categories (the top 10 GO categories in Fig. 4). Group 2 constitutes the intersection of genes possessing pattern 2 or 45 (hindbrain-activated genes) and genes involved in locomotory behavior. Curiously, most of those “forebrain-specific” genes are expressed not only in the forebrain but also in other areas or even the whole brain. Likewise, most of those “hindbrain-specific” genes are also expressed in other regions or the whole brain at certain time points. This non-local characteristic suggests that many genes may participate in distinct processes contingent on the locations and developmental stages.

### Dorsal-specific expressions are biased toward the early developmental stage

Figure 3 shows complex dependency between spatial expression patterns and their temporal occurrences. It is desirable to reduce this complex dependency into generic trends of spatial-temporal coupling if they exist. To fulfill this goal, we constructed four groups of spatial patterns according to a coarse-grained division: forebrain, hindbrain, dorsal and ventral. We then assigned genes to those four groups and evaluated the distributions of their occurrence time points. The evaluation procedures are described in Materials and Methods.

**Figure 6:**
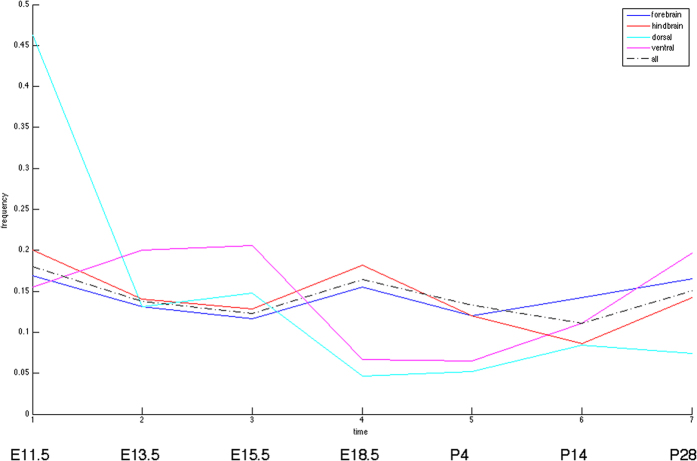
Temporal distributions of occurrences among the spatial expression patterns specific in the forebrain (blue), hindbrain (red), dorsal (cyan) and ventral (magenta) regions, as well as the background distribution of all patterns (black dashed line).

Figure 6 displays the distributions of occurrence times in those four groups and the background distribution among all reported spatial patterns. The temporal distributions of the forebrain and hindbrain groups are indistinguishable from the background. However, the occurrence of dorsal group patterns concentrates disproportionally at E11.5: about 46% of the dorsal patterns occur at E11.5, more than twice as the background share (about 18%) and about thrice as the ventral group share (about 16%) at E11.5. Conversely, the dorsal group has excessively low proportions at E18.5 and post-natal stages compared to the background. This trend is also observed by directly assigning level 5 substructures to those four groups. The temporal occurrence distributions of the four groups constructed from level 5 substructures are shown in Supplementary Figure S3.

### Genes sharing similar spatial-temporal expressions with *Lhx2* are differentially expressed in *Lhx2* conditional knockout mouse forebrain

A major hypothesis in functional genomics is that genes sharing similar expression profiles are linked by regulatory and functional interactions. To test this hypothesis in the spatial-temporal expression data of mouse brain development, we examined the changes of gene expression upon the deletion of *Lhx2*. Transcription factor *Lhx2* was selected as it is a key regulator for cortical development. In *Lhx2* null embryos where *Lhx2* is deleted from the beginning of development, cortex fails to form^47^. *Lhx2* was shown to regulate many forebrain development processes such as regional patterning^48^ and the proliferation of cortical progenitors^49^. Due to the significant phenotypes found in *Lhx2* knockout animals, it is proposed that *Lhx2* acts at the top of genetic pathways to regulate cortical development. Thus, we expect to see major changes in gene expression profile when *Lhx2* is deleted in the developing cortex.

We deleted *Lhx2* in the forebrain by crossing the Emx1-cre with mice carrying *Lhx2* floxed alleles. We collected dorsal telencephalon from E13.5 WT (wild type) and *Lhx2* cKO (*Lhx2*^f/f^; *Emx1*-Cre) mouse embryos and probed their transcriptomes with RNAseq. The 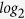 ratios of 17461 genes between the *Lhx2*-knock-out samples and normal controls were reported. The experimental and data processing procedures are described in Materials and Methods.

**Figure 7:**
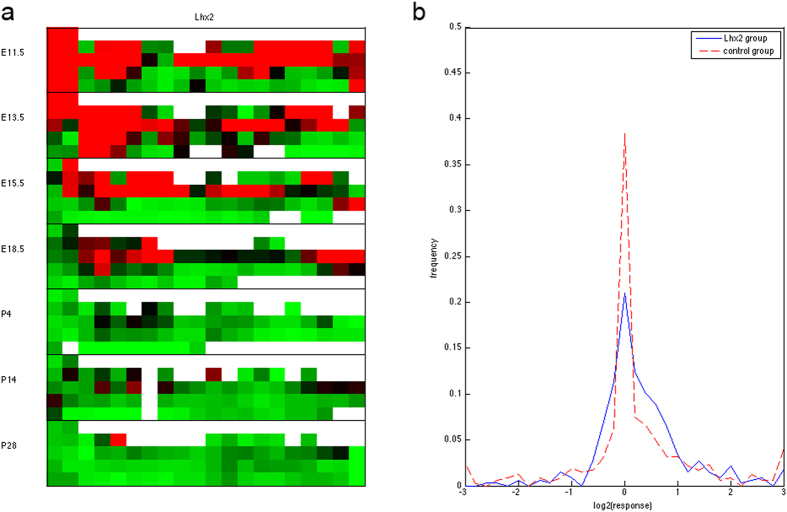
Genes co-expressed with *Lhx2* are more likely to be affected by the deletion of *Lhx2*. (**a**) Spatial-temporal expression profiles of *Lhx2* in the ABA data. The grid orientation and color code follow Fig. 3. (**b**) Distributions of 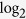 (forebrain tissue expression ratios) between the *Lhx2* 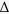 mutant and the wildtype control among the gene groups possessing similar (blue solid line) and opposite (red dashed line) expression patterns of *Lhx2* in the forebrain.

Figure 7a shows the *Lhx2* expression profiles in the ABA data. It is widely active in the forebrain and other regions at early developmental stages (E11.5, E13.5 and E15.5), becomes restricted to the alar layer of the posterior regions of the forebrain and the hindbrain at E18.5, and is silent at post-natal stages. We selected two groups of genes according to their similarity to *Lhx2* expression patterns: the *Lhx2* group consists of genes which are expressed in at least one substructure of the forebrain at the four prenatal stages, the control group consists of genes which are not expressed in any forebrain substructure at prenatal stages. These two groups comprise similar numbers of genes (328 for the *Lhx2* group and 469 for the control group).

Figure 7b displays the distributions of 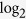 (expression ratios) among the two groups. Strikingly, compared to the control group, the *Lhx2* group genes exhibit a wider range of response variations. For instance, 28.78% of the *Lhx2* group genes yield 13.86% up or down regulation (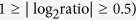, while only 21.34% of the control group genes yield the same level of responses. In contrast, the responses of the control group genes are highly concentrated around 0: 38.38% of the control group genes have 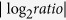 values smaller than 0.1, whereas 21.04% of the *Lhx2* group genes fall in this range. The p-value of the two-tailed Kolmogorov-Smirnov test was 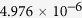. Curiously, deleting *Lhx2* induces more up-regulation responses than down-regulation responses among the *Lhx2* group (203 vs 97), the control group (218 vs 118), and the entire transcriptome (9991 vs 7470). The normalized RNAseq data of 17461 genes are reported in Supplementary Dataset S5.

Among the genes in the *Lhx2* group, we found many of them play important roles in cortical development. For example, the transcription factor Pax6 was shown to be involved in regulating cortical neurogenesis and patterning^50^ and Pax6 is reportedly a direct downstream target for *Lhx2*^51^. Additionally, in the *Lhx2* group, we found other transcription factors shown to regulate cortical neurogenesis, such as, Eomes/Tbr2^52^ and Ngn2^53^, and neural fate specification, such as Foxg1^54^, Cult2/Cux2^55^, and Fezf2^56^. Further, *Lhx2* group also consisted of genes functioning in neuronal migration and axon guidance, such as, members of Robo and Slit protein family^57^, Eph and Ephin protein family^58^ and Semaphorin/Nrp/Plxn protein families^59^. There are also members in several signaling pathways involved in cortical development, such as Wnt signaling pathway (such as, Apc, Ctnnb1, Gsk3b, Fzd), FGF signaling pathway (such as, Fgf1, Fgf15 and Fgfr1) and the Notch signaling pathway (such as, Notch1, Notch2, Hes5).

## Discussion

Gene expressions in developing mouse brain exhibit high level of spatial heterogeneity and temporal variation. In the spatial dimension, we reported the recurrent expression patterns that covered either globally (in most regions of the brain) global or locally (only in specific regions). In the temporal dimension, many expressed regions are time-varying. In addition, the spatial and temporal patterns are intertwined and gene-specific. Very few genes possess the same global or local spatial patterns over all time points (i.e., they are non-stationary). Conversely, many genes are neither globally expressed nor globally silent at all time points (i.e., they are heterogeneous). The relative scarcity of those decoupled patterns indicates that the “expression field”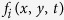 is not simply a tensor product of spatial and temporal functions 
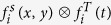
. Instead, it is likely a combination of multiple such “spatial-temporal modes”: 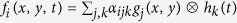.

Abundance of genes with no global expression states at any time point is expected, as many genes involved in brain development are expressed in specific regions rather than the entire brain. However, it is puzzling to observe 235 genes exhibiting global expressions at all the seven time points (Table 1). A possible explanation is that those genes are involved in the “house-keeping” functions required for the entire brain at all developmental stages.

We also observed the dearth of early/middle embryonic stage expressions (E11.5 , E13.5, E15.5) among the top-ranking global expression states (Table 1). This observation suggests that most genes probed in ABA are either silent or heterogeneously expressed in embryonic mouse brains. Spatial heterogeneity of gene expressions at early time points seems to contradict with the intuition that brains in the late developmental stage render more regional specialization and thus should be more heterogeneous. However, this observation agrees with the previous finding about the drop of regional specialization near birth^26^.

Functional enrichment of genes possessing each global expression state also reflects the trends of molecular events in mouse development. Figure 2 displays the enrichment outcomes in four broad functional classes. Genes involved in neural information processing (neural transmitters and receptors, signaling proteins) are enriched with the non-globally expressed states in the early/middle embryonic stages. This is consistent with the fact that neural signal transmission is not yet active at early developmental stages. In contrast, genes involved in the nervous system development seem to have different enriched global expression states than genes involved in the general system development. Transcription factors tend to be enriched in the global expression states with ones at late embryonic or post-natal stages. This observation coincides with the second wave of transcription factor activities as previously reported^3^.

The match between the locations of some recurrent local expression patterns and the enriched functions of their constituent genes verifies the links between expressions and functions of genes in brain development. Figure 4 demonstrates such links in three regions: forebrain (patterns 3 and 27) and forebrain/cerebral cortex development; medullary and pontomedullary (patterns 2 and 45) and locomotory behavior; and basal ganglia (pattern 8) and dopamine metabolism.

Despite the links between locations of spatial expression patterns and the functions of their constituent genes, we found only a limited number of genes are consistently expressed in a fixed region. Most genes consist of multiple expression patterns at each time point and different combinations of spatial patterns between distinct time points. Figure 5 gives several examples. This observation again implies that a gene is likely active in multiple regions at multiple developmental stages, depending on the expressions of other genes.

The coupling between spatial and temporal patterns of gene expressions is illustrated in Fig. 3. While some spatial patterns (such as patterns 1 and 2) occur uniformly at most time points (possibly in different genes), others (such as patterns 5 and 6) are biased toward a few time points. However, by assigning patterns into binary groups along the two axes on the sagittal plane (anterior-posterior, dorsal-ventral), we found only the dorsal region exhibits a strong bias at E11.5 (Fig. 6). Other regions showed no significant difference from the background in terms of temporal bias. The results suggest that spatial-temporal coupling likely occurs at a finer scale.

The RNAseq data comparing gene expression profiles between wild type and *Lhx2* conditional knock-out dorsal telencephalon in Fig. 7 indicate that genes sharing similar spatial-temporal expression patterns of *Lhx2* are differentially regulated relative to the genes without such patterns. However, the directions of differential expression become ambiguous, as there are higher fractions of both up and down regulated genes relative to the control experiments. While those directions can be explained by direct positive or negative regulation from *Lhx2* to its targets, they may also be attributed to indirect effects of the regulatory system. A down-regulated gene in *Lhx2* mutant may participate in the same functional pathway of *Lhx2* and co-regulated by the same genetic programs. Depletion of *Lhx2* disables (or activates) common regulators through a feedback link and shuts down the entire pathway. Conversely, an up-regulated gene in *Lhx2* mutant may possess a similar but complementary function of *Lhx2*. Depletion of *Lhx2* activates the complementary pathway and up-regulates the constituent genes.

The present study has several limitations. First, the rectangular grid of level 5 substructures greatly simplifies the problem but also loses information and introduces distortion. Fine-grained spatial variations are averaged out and thus possibly diluted or discarded. Furthermore, although the topology of the substructure layout remains largely invariant, the geometry undergoes substantial changes in development (http://developingmouse.brain-map.org/). Hence the geometric features in the grids such as distances between substructures are not accurate. Second, the spatial-temporal patterns of gene expressions are a concise, phenomenological representation of the observed data rather than a mechanistic model for brain development. A reasonable mechanistic model should specify both a network of neural (or regional) connections in the brain and a network of gene regulation. Such models are yet to be established. Third, we assumed brain substructures and gene expressions are symmetric between left and right brains and did not explore variations along the z axis. Fourth, we adopted binary quantization and discarded continuous expression values once the expressed regions were demarcated. Despite these shortcomings, the current decomposition of spatial-temporal patterns serves as a powerful template to investigate further questions regarding brain development.

## Materials and Methods

### ABA data processing

ABA provides *in situ* hybridization (ISH) data of gene expressions and their spatial-temporal locations in developing mouse brains^3^. The raw data constitute ISH section images annotated with the developmental time points and section coordinates. These raw data were summarized and transformed at several levels. First, a reference map of mouse brain at each developmental time point was constructed, and a coordinate transformation matrix from each ISH image to the corresponding reference map was provided. Second, the ISH section images of the same gene and brain sample were stacked together to form the corresponding 3D expression data file. Third, for each voxel in a 3D expression file, the density (fraction of expressing pixels in the voxel), intensity (the average fluorescence intensity of the expressing pixels in the voxel), and energy (density × intensity) were calculated. Fourth, a mouse brain was partitioned into substructures according to 10 nested ontological levels. Increasing-level ontologies represent more complex and later subdivision of brain structures. Detailed description of data specification is reported in the ABA website (help.brain-map.org/display/devmouse/Documentation).

We downloaded from the ABA website the raw ISH images and their summary data files. There were totally 19371 image-series covering 2005 genes and 7 developmental time points (E11.5, E13.5, E15.5, E18.5, P4, P14 and P28). The numbers of images and genes were smaller than those reported^3^, as we downloaded an earlier version of the data prior to the publication of the reference paper. We converted the XML data files into sparse matrices and calculated the average energy of each level 5 substructure for each gene at each time point. Furthermore, genes with missing values in all level 5 substructures were discarded. 1826 genes were selected for analysis. The data processing procedures are elaborated in the Supplementary Information.

### Demarcating expressed regions

For each gene 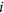 at each time point 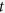, it is of interest to demarcate the contiguous regions where the expressions are elevated. The simplest method is to quantize 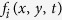 into binary values – 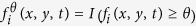 – and report contiguous patches where 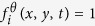. However, there is little or no prior knowledge about the threshold value 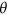. We adopted an approach from computational topology to determine 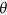. We sorted all distinct values of 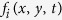 and varied 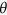 in the sorted values. For each fixed 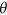, we generated 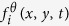, identified the contiguous components where 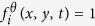, and counted the number of expressed components. Each 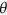 value gives rise to a component structure. Increasing 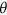 may increase the number of components by removing the bridges connecting two smaller components or reduce the number of components by removing components with lower expression levels. Accordingly, we can visualize the evolution of component structures by plotting 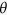 versus the corresponding number of components (Fig. 1c). Each component structure has a “valid interval” in terms of 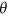. Within this interval the topology of the component structure (i.e., the number of components) does not change, although the component sizes can vary. A reasonable choice of the component structure is the most stable one with the longest valid interval of 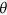. This component structure will suffer the least from 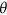 variation as its topology remains the invariant in the longest interval. Here we measured an interval length in terms of the number of distinct 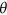 values within it rather than its geometric length. The quantization threshold 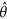 is the lower bound of the valid interval of the selected component structure. Moreover, to avoid choosing a very small 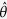 we required the lower bound of the selected interval to exceed a threshold (0.2). In the example of Fig. 1, the selected threshold value 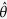 was the lower bound of this interval (0.2384 in Fig. 1c). The expressed regions were determined by substituting 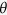 in the quantization function (Fig. 1d). Choosing the lower and upper bounds of the valid interval generates the largest and smallest expressed patches of the same component structure. The lower bound captures the largest possible areas which are differentially expressed relative to the remaining substructures. In contrast, the upper bound highlights only the areas with the highest expression levels. The difference between the choice of lower and upper bounds is illustrated in Supplementary Figure S4. Suppose the expression level of a gene has two overlapped bumps along a one-dimensional grid (upper left). By varying the threshold value from the lowest to the highest expression level, we found that two components yielded the longest threshold interval (upper right). Choosing the lower bound of this interval yields two large bumps separated by an infinitesimal distance (lower left, two red bumps). Instead, choosing the upper bound of this interval yields two points at the peaks of the two bumps (lower right, two red dots).

The aforementioned quantization procedure can demarcate expressed regions relative to other substructures at the same time point, but does not directly compare expression data across time points. Images generated from different mice at the same time point can be normalized according to a reference transformation provided by ABA. In contrast, temporal comparison is much more challenging since the mapping between regions of different time points results from the complicated developmental process and is not provided in the database. Therefore, we did not attempt to compare continuous expression levels between time points directly. Instead, with our demarcation method, we can track the quantized expression dynamics of each substructure by checking whether it is expressed or unexpressed relative to other substructures over all time points.

The quantization procedure is robust against data normalization procedures such as z-scores, rank transform^60^, PaGeFinder^61^, PaGenBase^62^, and TiSGeD^63^ since the threshold was obtained from the sorted data point values and the order of data point values is preserved in most normalization procedures.

### Constructing and sorting global expression states

For gene 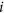 at time point 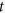, we determined whether 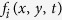 is globally expressed by checking whether the largest component derived from 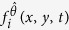 contains more than half of the level 5 substructures with valid data. This procedure is justified by randomized experiments. Given a probability 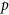, we generated 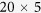 binary random matrices by sampling each entry with 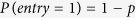. The size of the largest connected component in the random matrices follows a Poisson distribution (Supplementary Figure S5). By varying 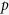 from 0.4 to 0.9, we found the probability that the largest component arising from random matrices exceeds half of the matrix size is very small.

The aforementioned procedure 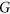 maps 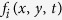 into a binary value. For gene 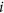, the global expression state 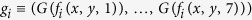 is a 7-bit binary vector indicating the global expression status at each time point. There are totally 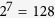 global expression states. We counted the number of genes belonging to each state and sorted the states in a decreasing order.

### Identifying and ranking spatial expression patterns

In addition to global expression states, we also wanted to extract the recurrent local expression patterns. A recurrent local expression pattern is defined as a collection of overlapping and contiguous areas which are demarcated as expressed regions in multiple gene-time point combinations. The algorithm of identifying the recurrent local expression patterns is described with more details in Supplementary Information. Briefly, for each gene-time point combination, we extracted the contiguous expressed regions (a.k.a. components), selected them with several filtering criteria, and applied a graph theory based algorithm^60^ to cluster the components. The outputs of the algorithm are clusters of overlapped contiguous regions. Each cluster corresponds to a recurrent local expression pattern. For each spatial expression pattern, we counted the number of occurrences among all genes at all time points, and sorted the spatial expression patterns according to their counts.

The Matlab programs of quantizing the expression data, generating global expression states and recurrent local expression patterns are provided in Supplementary Information. The input data for the Matlab programs are provided in Supplementary Dataset S6.

### Evaluating functional enrichments of genes possessing each global expression state or spatial expression pattern

We extracted genes possessing each global expression state and spatial expression pattern. Hypergeometric enrichment p-values of GO categories with multiple-hypotheses correction in these groups of genes were evaluated by the online bioinformatic tool DAVID^64^. All enrichment outcomes reported by DAVID appear in Supplementary Datasets S2 and S4.

### Evaluating the temporal distributions of forebrain, hindbrain, dorsal and ventral specific expressions

Among the 45 spatial expression patterns we identified the ones which were active primarily in the forebrain (3, 4, 6, 8, 10, 11, 16, 18, 27, 31, 32, 34, 36, 37, 38), hindbrain (1, 2, 5, 7, 9, 13, 14, 15, 17, 19, 20, 21, 22, 24, 26, 30, 35, 43, 45), dorsal (5, 11, 14, 19, 27, 32, 34) and ventral (10, 13, 16, 17, 18, 20, 21, 24, 26, 30, 31, 35, 39) regions. The control group comprised all 45 patterns. In each group, we extracted the expressed regions belonging to the constituting patterns and evaluated the distribution of their temporal occurrences. We also discarded spatial patterns and simply considered the expressed regions whose coordinates fell within the ranges of the five groups (forebrain: 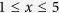, hindbrain: 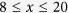, dorsal: 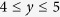, ventral: 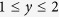, control: all coordinates). The temporal occurrence distributions of the five groups were again evaluated.

### Processing and analyzing the RNAseq data under the *Lhx2*-knockout treatment

The generation of *Lhx2* cKO was described previously^42^. We isolated total RNA from dorsal telencephalon from E13.5 control (*Lhx2*^f/f^) and cKO (*Lhx2*^f/f^; Emx1-Cre) with Trizol (Invitrogen) according to the manufacturer’s protocol. The quality and integrity of the collected RNA was evaluated with the 2100 Bioanalyzer RNA Nano Chip (Agilent). RNA from three control and cKO dorsal telencephalons was analyzed by RNA-Seq. Aligned reads were calculated as reads per kilobase of exon model per million mapped reads (RPKM).

We selected two groups of genes according to their similarity to the *Lhx2* spatial-temporal gene expression from the ABA data. The *Lhx2* group consists of 328 genes which are expressed in at least one substructure of the forebrain at the four prenatal stages. The control group consists of 469 genes which are not expressed in any forebrain substructure at prenatal stages. We evaluated the log2 ratio of the expressed values of each gene between the *Lhx2* knock-out mutant and the wild type. The distributions of log2 ratios of the two groups of genes were compared visually (Fig. 7b) and by the Kolmogorov-Smirnov test.

## Additional Information

**How to cite this article**: Chou, S.-J. *et al*. Analysis of spatial-temporal gene expression patterns reveals dynamics and regionalization in developing mouse brain. *Sci. Rep.* **6**, 19274; doi: 10.1038/srep19274 (2016).

## Supplementary Material

Supplementary Information

Supplementary Table 1

Supplementary Table 2

Supplementary Table 3

Supplementary Table 4

Supplementary Table 5

Supplementary Table 6

## Figures and Tables

**Table 1:** Numbers of genes possessing each top-ranking global expression state.

E11.5	E13.5	E15.5	E18.5	P4	P14	P28	# genes
0	0	0	0	0	0	0	453
1	1	1	1	1	1	1	235
0	1	1	1	1	1	1	86
0	0	0	1	1	1	1	79
0	0	0	0	0	1	0	76
0	0	0	0	0	1	1	73
0	0	0	0	1	1	1	62
0	0	1	1	1	1	1	61
0	0	0	0	1	1	0	41
0	0	0	0	1	0	0	39
0	0	0	1	1	1	0	30
0	0	0	1	0	0	0	29
1	0	1	1	1	1	1	26
0	0	0	1	1	0	0	25
0	0	0	1	0	1	0	22
1	1	1	0	1	1	1	22
0	0	0	0	0	0	1	21
0	0	0	1	0	1	1	20
1	0	0	1	1	1	1	19
1	1	1	1	1	1	0	19
1	0	0	0	1	1	1	15
1	1	1	1	0	1	1	15
0	0	0	0	1	1	1	14
0	0	0	1	1	1	1	14
1	0	0	1	1	1	1	14
1	0	0	0	0	0	0	13
0	1	1	1	1	1	0	12
0	0	0	0	0	0	0	11
1	0	0	0	0	1	0	11
