# Supplementary material for: Analysis of spatial-temporal gene expression patterns reveals dynamics and regionalization in developing mouse brain
Source: Sci Rep. 2016 Jan 20;6:19274. doi: 10.1038/srep19274 (PMC4726224; doi:10.1038/srep19274)
Supplement: Supplementary Information [file srep19274-s1.pdf]

Supplementary Information: Analysis of spatial-temporal gene expression patterns reveals dynamics and regionalization in developing mouse brain

Shen-Ju Chou[2], Chindi Wang[1], Nardnisa Sintupisut[1], Zhen-Xian Niou[2], Chih-Hsu Lin[1], Ker-Chau Li[1], and Chen-Hsiang Yeang[1]

1. Institute of Statistical Science, Academia Sinica, Nankang, Taipei, Taiwan
2. Institute of Cellular and Organismic Biology, Academia Sinica, Nankang, Taipei, Taiwan

Supplementary file information

Supplementary Information: The ABA data processing procedures and the algorithm of finding local gene expression patterns. Supplementary Figures. The Matlab program source codes of analyzing ABA data.

Supplementary Dataset S1: The genes belonging to each global expression state.

Supplementary Dataset S2: Complete enrichment outcomes of GO categories.

Supplementary Dataset S3: The genes possessing each local pattern.

Supplementary Dataset S4: The complete GO category enrichment outcomes of spatial patterns.

Supplementary Dataset S5: The normalized RNAseq data of 17461 genes in the forebrain of Lhx2 knock-out mice.

Supplementary Dataset S6: The input data for the Matlab programs.

Allen Developing Mouse Brain Atlas (ABA) data preprocessing

ABA provides *In Situ* hybridization (ISH) data including an anatomic structure and a heatmap representation of gene expression for an experiment or a gene in 3D format (Lein et al., 2007). An individual ISH section images for each time-point are stacked into a single slide image to form one common space called 3D reference model. A position of each section in the brain or specimen is calculated to a reference section index across all time-points. Each ISH image is masked with grids to create a 3D summary of the gene expression and project the data to a common coordinate space of the 3D reference model to enable spatial comparison between data from different specimens. The resolution of the data grids varies with age and corresponds with the sampling density for that time-point. All images and quantified expression values by structure or as 3D grids including the annotation of the voxels are available for download via API service (http://help.brain-map.org/display/devmouse/API). In this study, we collected the quantified expression values as 3D grids. There are four steps to retrieve and parse the data form ABA. The whole data was automated under Linux environment.

1. We tracked a list of all gene and image-series IDs available on ABA from gene detail pages (see a prototype and example from Table 1). The data including gene identifiers, specimen related information (i.e. age) and meta-information for each image (i.e. path, dimension) comprising the image-series are available as XML format. The XML header shows a total number of genes and pages. There are 2005 genes and 19371 image-series IDs for 7 developmental ages (E11.5, E13.5, E15.5, E18.5, P4, P14 and P28). Note that the latest data has been updated in June, 2013. All XML files are zipped in ABA_xml.zip file. Java code written in ABA_xml_processing.java file is applied to parse the XML data. The summary gene and image-series information are summarized in aba_dev_allgenes_20120424.2.txt file with tab delimited format.

**Prototype**	http://developingmouse.brain-map.org/data/search/gene/index.xml?page=[i]&term=*<br><br>where [i] is the page number ranging from 1 to 201.
**Return type**	XML file.
**Comments**	Each markup starting with tag gene contains gene and image series information. One gene ID

	can contain more than one age. One age contains one image series ID.

Example

http://developingmouse.brain-map.org/data/search/gene/index.xml?page=1&term=*


```xml
<genes total-entries="2005" current-page="1" total-pages="201" per-page="10">
  <gene>
    <chromosome>14</chromosome>
    <chromosome-id>40</chromosome-id>
    <created-at>2004-09-18T14:43:13-07:00</created-at>
    <entrez-id>278672</entrez-id>
    <gene-symbol>1110051B16Rik</gene-symbol>
    <homologene-id>18848</homologene-id>
    <id>93453</id>
    <name>RIKEN cDNA 1110051B16 gene</name>
    <organism>Mus musculus</organism>
    <organism-id>2</organism-id>
    <project-code>0350</project-code>
    <updated-at>2009-01-29T17:05:50-08:00</updated-at>
    <image-series type="array">
      <image-series>
        <age>E11.5</age>
        <created-at>2009-07-01T16:10:35-07:00</created-at>
        <days>11.5</days>
        <donor-id>9215</donor-id>
        <expression>true</expression>
        <failed>false</failed>
        <gene-id>93453</gene-id>
        <id>100073527</id>
        <is-experiment>true</is-experiment>
        <isembryonic>1</isembryonic>
        <medical-condition>TS19</medical-condition>
        <name>100073527</name>
        <organism>Mus musculus</organism>
        <plane-of-section>sagittal</plane-of-section>
        <project-code>0350</project-code>
        <published-at>2009-10-01</published-at>
        <qc-date>2009-07-16T12:54:17-07:00</qc-date>
        <race-or-strain>C57BL/6J</race-or-strain>
        <run-group-id>707117</run-group-id>
        <run-group-type>full set</run-group-type>
        <runplan-id>656</runplan-id>
        <sex>unknown</sex>
        <specimen-id>700715</specimen-id>
        <storage-directory>/external/devmouse/prod170/image_series_100073527/</storage-
        directory>
        <trv>...</trv>
        <tvr>...</tvr>
        <updated-at>2010-02-13T19:48:19-08:00</updated-at>
      </image-series>
      <image-series>...</image-series>
      <image-series>...</image-series>
      <image-series>...</image-series>
      <image-series>...</image-series>
      <image-series>...</image-series>
      <image-series>...</image-series>
      <image-series>...</image-series>
    </image-series>
  </gene>
  <gene>...</gene>
  <gene>...</gene>
  <gene>...</gene>
  <gene>...</gene>
  <gene>...</gene>
  <gene>...</gene>
  <gene>...</gene>
  <gene>...</gene>
  <gene>...</gene>
</genes>
```

2. We further downloaded the 3D expression summaries of each image-series ID. There are 15531 and 5840 IDs with- and without 3D grid data files, respectively.

**Prototype**	1. http://developingmouse.brain-map.org/grid_data/download/[ImageSeriesID]<br>2. http://api.brain-map.org/grid_data/download/[ImageSeriesID]	
**Return type**	1. A zip file containing 5 files including image_series.xml, density.mhd, density.raw, intensity.mhd and intensity.raw.<br>2. A zip file containing 3 files including data_set.xml, energy.mhd and energy.raw.	
	**image_series.xml**<br>**data_set.xml**	XML file containing information about the specimen and images. This file is returned if the volumes parameter is null.
	**density.raw**	A raw uncompressed float (32bit) little-endian volume representing average expression density per voxel.<br>Volume dimension is dependent on specimen age. Value of "-1" represents no data.
	**intensity.mhd**	A simple text header file in MetaImage format describing the volume.
	**intensity.raw**	A raw uncompressed float (32bit) little-endian volume representing average expression intensity per voxel.<br>Volume dimension is dependent on specimen age. Value of "-1" represents no data.
	**energy.mhd**	A simple text header file in MetaImage format describing the volume. This file is returned if the volumes parameter is null.
	**energy.raw**	A raw uncompressed float (32-bit) little-endian volume representing average expression energy per voxel. A value of "-1" represents no data. This file is returned if the volumes parameter is null.
**Comments**	**expression density**	Expression density is the number of expressing pixels divided by the number image pixels overlapping the voxel. A value of "-1" indicates no data. This value represents the size of the sphere at each grid location.
	**expression intensity**	Expression intensity is the averaged inverted ISH grayscale value at expressing pixels within the span of the grid voxel. A value of "-1"

		indicates no data. This value represents the color of the sphere at each grid location.
	**expression energy**	Expression energy is defined as (expression intensity * expression density). Anatomic search, temporal search, Neuroblast and AGEA features on the Atlas are all based on expression energy. A value of "-1" indicates no data.

Example

1. http://developingmouse.brain-map.org/grid_data/download/100073527

image-series.xml file

```
<image-series>
  <age>E11.5</age>
  <created-at>2009-07-01T16:10:35-07:00</created-at>
  <days>11.5</days>
  <donor-id>9215</donor-id>
  <expression>true</expression>
  <failed>false</failed>
  <gene-id>93453</gene-id>
  <id>100073527</id>
  <is-experiment>true</is-experiment>
  <isembryonic>1</isembryonic>
  <medical-condition>TS19</medical-condition>
  <name>100073527</name>
  <organism>Mus musculus</organism>
  <plane-of-section>sagittal</plane-of-section>
  <project-code>0350</project-code>
  <published-at>2009-10-01</published-at>
  <qc-date>2009-07-16T12:54:17-07:00</qc-date>
  <race-or-strain>C57BL/6J</race-or-strain>
  <run-group-id>707117</run-group-id>
  <run-group-type>full set</run-group-type>
  <runplan-id>656</runplan-id>
  <sex>unknown</sex>
  <specimen-id>700715</specimen-id>
  <storage-directory>/external/devmouse/prod170/image_series_100073527/</storage-directory>
  <trv>...</trv>
  <tvr>...</tvr>
  <updated-at>2010-02-13T19:48:19-08:00</updated-at>
  <gene>...</gene>
  <images type="array">...</images>
  <probes type="array">...</probes>
</image-series>
```

density.mhd

```
ObjectType = Image
NDims = 3
BinaryData = True
BinaryDataByteOrderMSB = False
CompressedData = False
TransformMatrix = 1 0 0 0 1 0 0 0 1
Offset = 0 0 -160
CenterOfRotation = 0 0 0
AnatomicalOrientation = RAI
ElementSpacing = 80 80 80
DimSize = 70 75 40
ElementType = MET_FLOAT
ElementDataFile = density.raw
```

intensity.mhd

```
ObjectType = Image
NDims = 3
BinaryData = True
BinaryDataByteOrderMSB = False
CompressedData = False
TransformMatrix = 1 0 0 0 1 0 0 0 1
Offset = 0 0 -160
CenterOfRotation = 0 0 0
AnatomicalOrientation = RAI
ElementSpacing = 80 80 80
DimSize = 70 75 40
ElementType = MET_FLOAT
ElementDataFile = intensity.raw
```

2. http://api.brain-map.org/grid_data/download/100073527

data_set.xml


```xml
<section-data-set>
  <expression>true</expression>
  <failed>false</failed>
  <id>100073527</id>
  <name nil="true"/>
  <qc-date>2009-07-16T12:54:17Z</qc-date>
  <reference-space-id>1</reference-space-id>
  <section-thickness>20</section-thickness>
  <specimen-id>700715</specimen-id>
  <storage-directory>/external/devmouse/prod170/image_series_100073527/</storage-directory>
  <weight>5200</weight>
  <genes>...</genes>
  <probes>...</probes>
  <products>...</products>
  <specimen>...</specimen>
  <alignment3d>...</alignment3d>
  <section-images>...</section-images>
  <plane_of_section>sagittal</plane_of_section>
  <age>E11.5</age>
</section-data-set>
```


energy.mhd

```
ObjectType = Image
NDims = 3
BinaryData = True
BinaryDataByteOrderMSB = False
CompressedData = False
TransformMatrix = 1 0 0 0 1 0 0 0 1
Offset = 0 0 -160
CenterOfRotation = 0 0 0
AnatomicalOrientation = RAI
ElementSpacing = 80 80 80
DimSize = 70 75 40
ElementType = MET_FLOAT
ElementDataFile = energy.raw
```

3. We applied MATLAB code (see process_read3Dfiles.m) to read the intensity, density or energy values, extract the coordinates corresponding to the expression values and visualize their 3D images from raw files (i.e. intensity.raw, density.raw and energy.raw).

**Input**	A pair of *.raw and *.mhd file. For instance, [density.raw and density.mhd] or [intensity.raw and intensity.mhd] or [energy.raw and energy.mhd].
**Output**	1. [expressionType]_ raw_[image-seriesID].coor<br>2. [expressionType]_[image-seriesID].coor<br>3. [expressionType]_[image-seriesID].png<br>where [expressionType] represents a file of interest (i.e. density, intensity or energy). A file with “coor” extension is the text file containing coordinates of image and the corresponding expression values. A “raw” tag means to a raw data from *.raw file. A file without “raw” tag represents a file containing normalized expression values. All output files were put into the same folder as input file. The voxel 3D coordinates are reconstructed in RAI orientation (+x = right, +y=anterior, +z= inferior). The expression values are normalized by applying the cumulative distribution function (CDF) value with the range [0, 1] to the entire matrix. A value of “0” indicates that coordinate is not valid. Only valid values are collected.
**Example**	**Input: [intensity.raw and intensity.mhd] of image-series ID 100073527.**<br>**Output:**<br>**1. intensity_raw_100073527.coor**<br>- A dimension size of 3D image is 70 x 75 x 40.

x	y	z	values
59	6	1	104.153831481934
60	12	1	106.769218444824
61	12	1	105.989677429199
60	13	1	106.020446777344
61	13	1	103.108413696289
62	13	1	86.1428604125977
63	13	1	86.1428680419922
64	13	1	86.1428680419922
60	14	1	105.230545043945
61	14	1	98.8087539672852
62	14	1	86.1428604125977
63	14	1	86.1428680419922
64	14	1	86.1428680419922
61	15	1	94.1960906982422
62	15	1	87.1479721069336
63	15	1	86.1428527832031
64	15	1	86.1428604125977
61	16	1	90.6747665405273
62	16	1	87.9549865722656
...	...	...	...

2. intensity_100073527.coor

x	y	z	values
59	6	1	0.54393116157709
60	12	1	0.551150243572653
61	12	1	0.548983566588498
60	13	1	0.549035947790312
61	13	1	0.541145434026033
62	13	1	0.515002452393033
63	13	1	0.51503102395766
64	13	1	0.51503102395766
60	14	1	0.546864508878719
61	14	1	0.532326344411406
62	14	1	0.515002452393033
63	14	1	0.51503102395766
64	14	1	0.51503102395766
61	15	1	0.524073924161864
62	15	1	0.516202458107337
63	15	1	0.514992928538158
64	15	1	0.515002452393033
61	16	1	0.52018342944526
62	16	1	0.517045319263812
...	...	...	...

3. intensity_100073527.png

- The coordinates of axis Z from 1 to 37 contain valid expression intensity values.

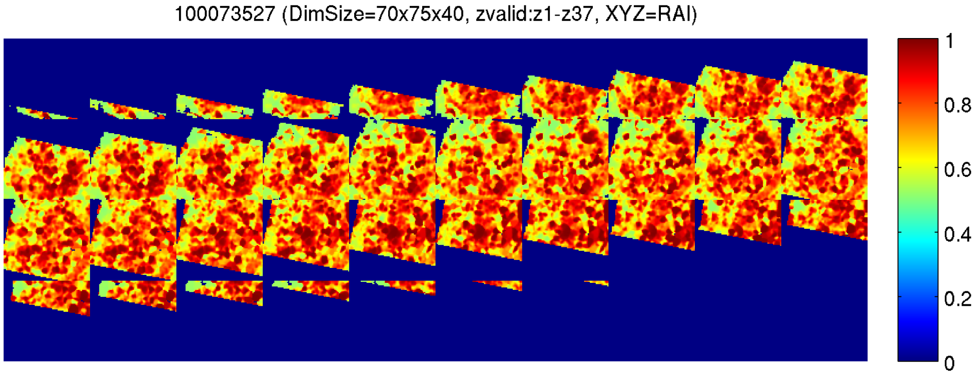

Structure Ontology Hierarchy

The structure ontology hierarchy was downloaded from the following webpage http://api.brain-map.org/api/v2/structure_graph_download/13.json. The json file was parsed and reformatted into a more readable tabular-form file ***brain_anno.csv*** for easier lookup of each structure with its annotation.

Mouse gene ontology

The document of GO terms for all mouse genes was downloaded from mouse genome informatics from the Gene Ontology Data section of the following website: ftp://ftp.informatics.jax.org/pub/reports/index.html#homology.

Algorithm of identifying the recurrent local expression patterns

1. For each gene at each time point, convert its expression data $f_i(x,y,t)$ into binary values $f_i^{\hat{\theta}}(x,y,t)$ according to the aforementioned quantization procedure.
2. Extract the contiguous regions where $f_i^{\hat{\theta}}(x,y,t)$=1 from all gene-time point combinations.
3. Select the contiguous regions that pass fiver filtering criteria: (1)they cover more than 2 substructures, (2)the corresponding $f_i(x,y,t)$is not labeled as globally expressed, (3)their sizes are less than one half of all substructures with valid data, (4)their sizes are at least 75% of the

sizes of their convex hulls, and (5)the numbers of substructures outside the regions and inside their convex hulls are smaller than 8. We name the contiguous regions that pass those criteria as local components. There are 3847 local components in the ABA data.

4. For each pair of components, we define their similarity as the ratio of the size of their intersection to the maximal size of the two components. Calculate the similarity matrix between all pairs of local components.
5. Apply a graph theory based clustering algorithm to the similarity matrix.
   - 5.1 Sort the similarity scores in a decreasing order.
   - 5.2 Initially set a graph of 3847 isolated nodes and the threshold value to the maximal similarity score. Repeat the following procedures:
     - 5.2.1 Lower the threshold to the next value in the ranked list.
     - 5.2.2 Add edges to the node pairs whose similarity scores exceed the current threshold.
     - 5.2.3 Expand and merge old cliques (maximally complete subgraphs) from previous steps with newly added edges.
     - 5.2.4 Find new cliques among the newly added edges.
     - 5.2.5 Stop when the current threshold value <= 0.5.
   - 5.3 Initially set each clique as a cluster. Repeat the following procedures:
     - 5.3.1 Sort clusters by their sizes.
     - 5.3.2 Pick two mergeable clusters with the largest sizes. Two clusters are mergeable if at least 70% of the inter-cluster edges have similarity score >= 0.4. Merge the chosen clusters.
     - 5.3.3 Stop when no cluster pairs are mergeable.
   - 5.4 Report the clusters with at least 10 members.

The outputs of the algorithm are clusters of overlapped contiguous regions. Each cluster corresponds to a recurrent local expression pattern.

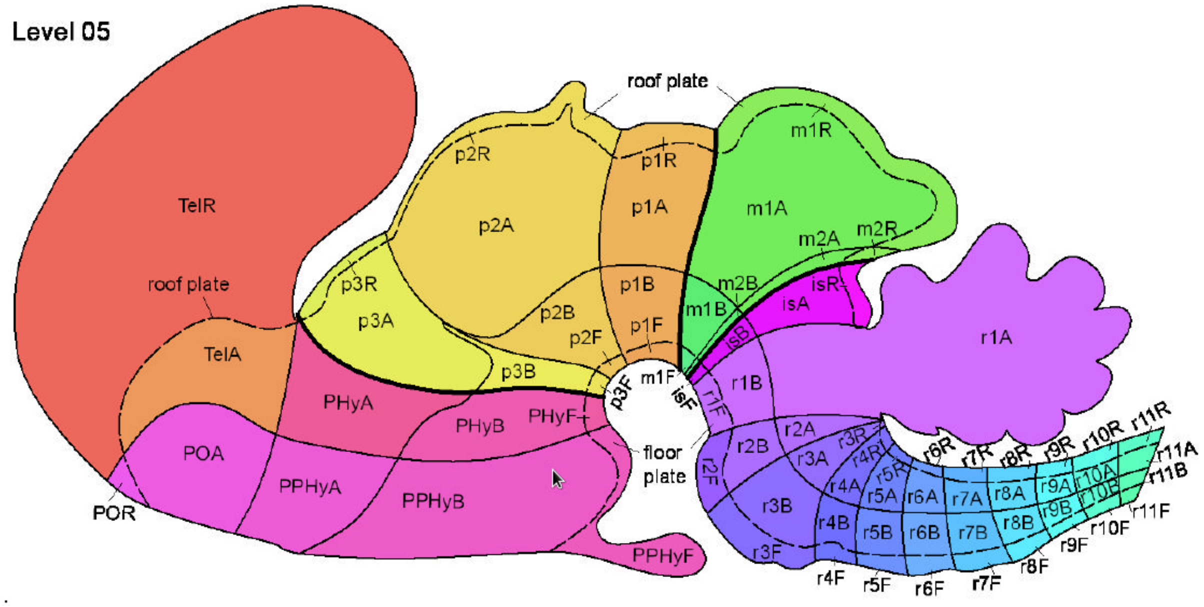

Supplementary Figure S1: Level 5 substructures of a mouse brain. Adapted from the ABA manual.

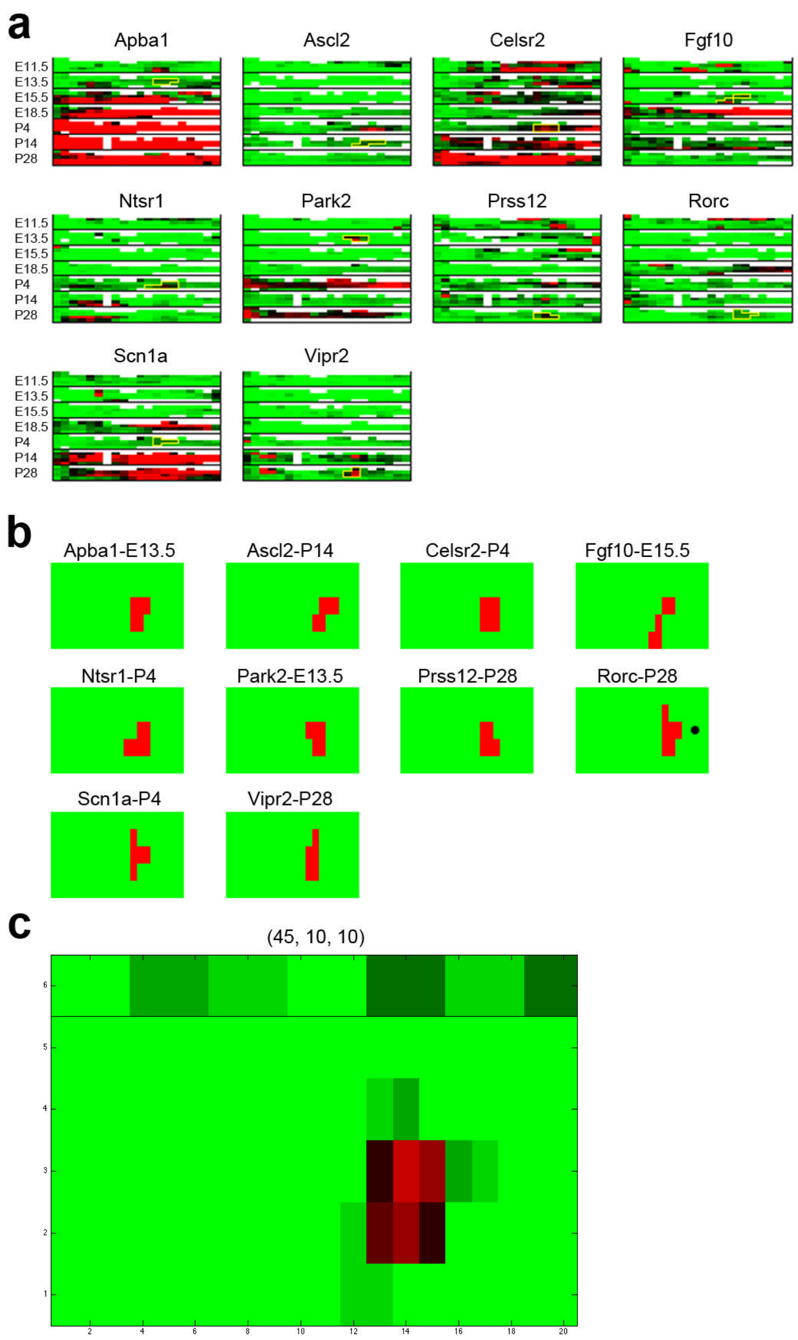

Supplementary Figure S2: An example of the recurrent local spatial expression pattern 45. The top panels display the expressions of the 10 genes possessing the pattern. The middle panels display the members of pattern 45 as expressed regions in the 10 genes. The bottom panel summarizes the occurrence frequency of each time point (the top bar) and substructure (the red rectangle) in the pattern.

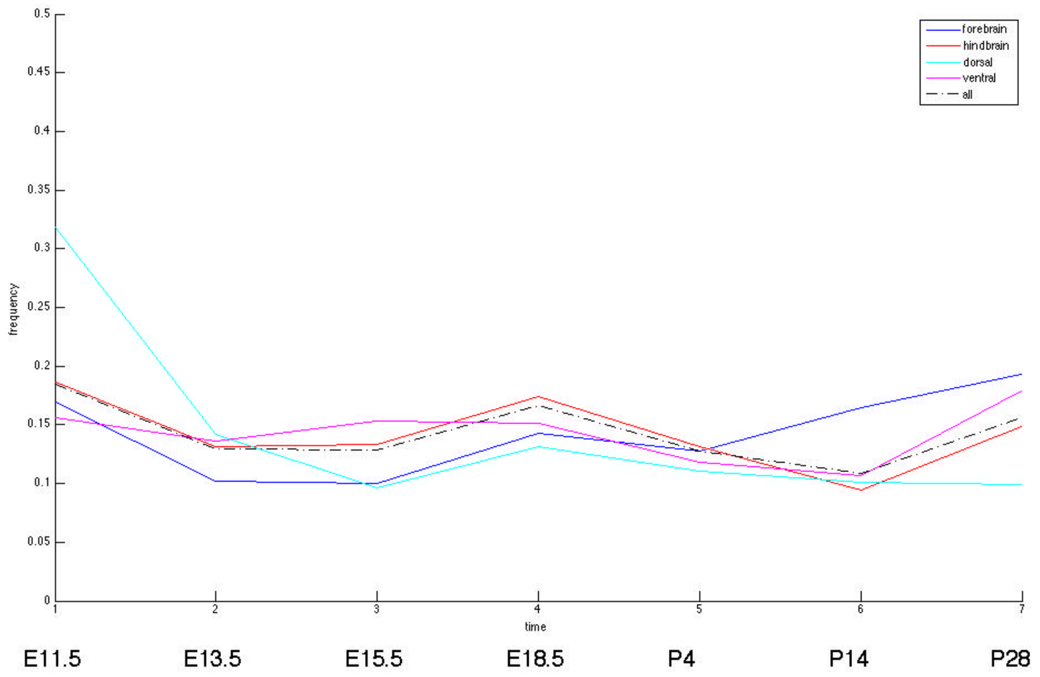

Supplementary Figure S3: The temporal occurrence distributions of the four groups constructed from level 5 substructures.

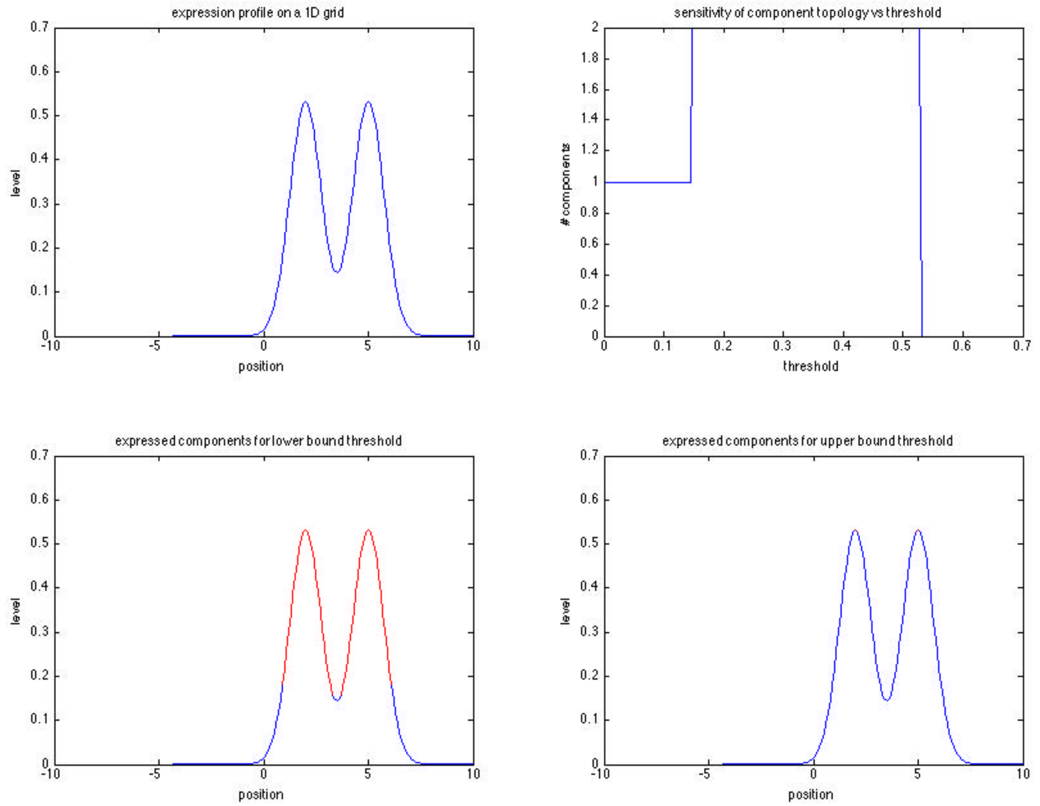

Supplementary Figure S4: A toy example illustrating the difference of choosing the lower and upper bounds in the valid interval as the threshold for expression quantization.

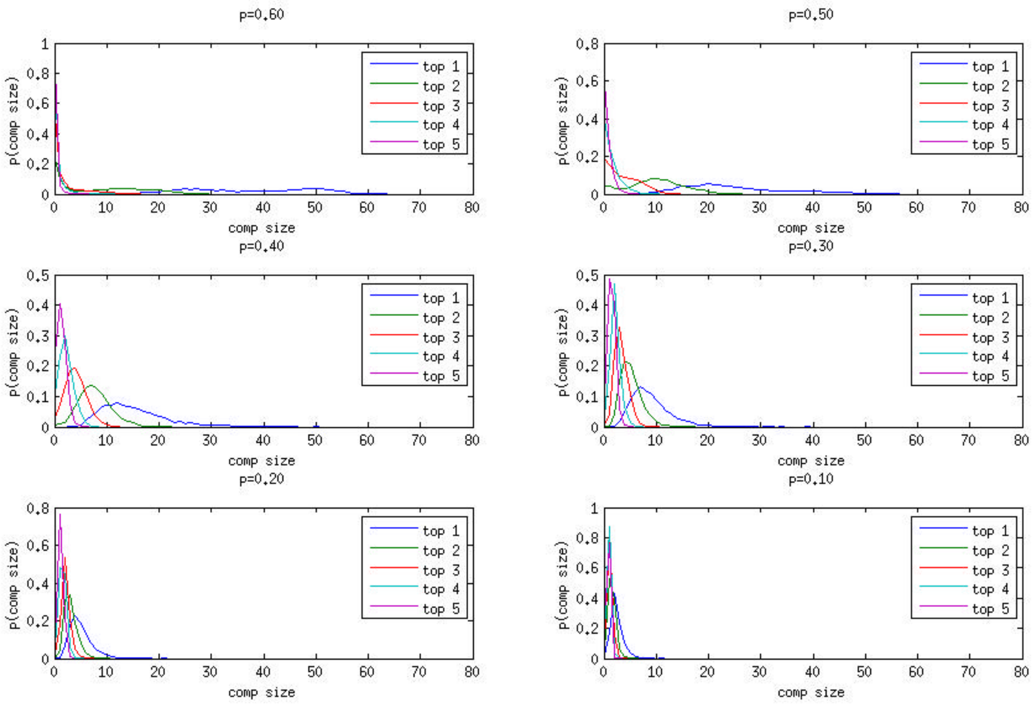

Supplementary Figure S5: The component size distributions derived from random binary matrices. Each panel shows the experiments with a decreasing probability of assigning 1 to the random matrix entries. In each panel, the size distributions of top 5 components are reported.

Matlab Program Source Codes for Analyzing the ABA Data

```
%%%%%%%%%%%%%%%%%%%%%%%%%%%%%%%%%%%%%%%%%%%%%%%%%%%%%%%%%%%%%%%%%%%%%%%%%%%
%%%%%%%%%%%%%%%%
% Matlab programs for analyzing the spatial-temporal ABA expression data.
%%%%%%%%%%%%%%%%%%%%%%%%%%%%%%%%%%%%%%%%%%%%%%%%%%%%%%%%%%%%%%%%%%%%%%%%%%%
%%%%%%%%%%%%%%%%


%%%%%%%%%%%%%%%%%%%%%%%%%%%%%%%%%%%%%%%%%%%%%%%%%%%%%%%%%%%%%%%%%%%%%%%%%%%
% ABAcodes_mainprogram.m.
%%%%%%%%%%%%%%%%%%%%%%%%%%%%%%%%%%%%%%%%%%%%%%%%%%%%%%%%%%%%%%%%%%%%%%%%%%%


%%%%%%%%%%%%%%%%%%%%%%%%%%%%%%%%%%%%%%%%%%%%%%%%%%%%%%%%%%%%%%%%%%%%%%%%%%%
% The main program for analyzing the spatial-temporal ABA expression data.
%%%%%%%%%%%%%%%%%%%%%%%%%%%%%%%%%%%%%%%%%%%%%%%%%%%%%%%%%%%%%%%%%%%%%%%%%%%

% The inputs of the program include the processed mouse brain gene
expression data, names of genes, names of substructures in the grid, and
names of the time points.
% The brain gene expression data is a four-dimensional array:
braindata(gene index,substructure along the anterior-posterior
axis,substructure index along the dorsal-ventral axis, time).
% The outputs are stored in a Matlab file designated by outputfilename.

% In the ABA data we analyzed, we use the following inputs.
%ngenes=1826; nx=20; ny=5; ntimesteps=7;
%gridnames{1}{1}='PPHyF'; gridnames{1}{2}='PPHyB';
gridnames{1}{3}='PPHyA'; gridnames{1}{4}='POA'; gridnames{1}{5}='POR';
%gridnames{2}{1}='PHyF'; gridnames{2}{2}='PHyB'; gridnames{2}{3}='PHyA';
gridnames{2}{4}='TelA'; gridnames{2}{5}='TelR';
%gridnames{3}{1}='p3F'; gridnames{3}{2}='p3B'; gridnames{3}{3}='p3A';
gridnames{3}{4}='p3R'; gridnames{3}{5}='';
%gridnames{4}{1}='p2F'; gridnames{4}{2}='p2B'; gridnames{4}{3}='p2A';
gridnames{4}{4}='p2R'; gridnames{4}{5}='';
%gridnames{5}{1}='p1F'; gridnames{5}{2}='p1B'; gridnames{5}{3}='p1A';
gridnames{5}{4}='p1R'; gridnames{5}{5}='';
%gridnames{6}{1}='m1F'; gridnames{6}{2}='m1B'; gridnames{6}{3}='m1A';
gridnames{6}{4}='m1R'; gridnames{6}{5}='';
%gridnames{7}{1}='m2F'; gridnames{7}{2}='m2B'; gridnames{7}{3}='m2A';
gridnames{7}{4}='m2R'; gridnames{7}{5}='';
%gridnames{8}{1}='isF'; gridnames{8}{2}='isB'; gridnames{8}{3}='isA';
gridnames{8}{4}='isR'; gridnames{8}{5}='';
%gridnames{9}{1}='r1F'; gridnames{9}{2}='r1B'; gridnames{9}{3}='r1A';
gridnames{9}{4}=''; gridnames{9}{5}='';
%gridnames{10}{1}='r2F'; gridnames{10}{2}='r2B'; gridnames{10}{3}='r2A';
gridnames{10}{4}=''; gridnames{10}{5}='';
%gridnames{11}{1}='r3F'; gridnames{11}{2}='r3B'; gridnames{11}{3}='r3A';
gridnames{11}{4}='r3R'; gridnames{11}{5}='';
%gridnames{12}{1}='r4F'; gridnames{12}{2}='r4B'; gridnames{12}{3}='r4A';
gridnames{12}{4}='r4R'; gridnames{12}{5}='';
```

```
%gridnames{13}{1}='r5F'; gridnames{13}{2}='r5B'; gridnames{13}{3}='r5A';
gridnames{13}{4}='r5R'; gridnames{13}{5}='';
%gridnames{14}{1}='r6F'; gridnames{14}{2}='r6B'; gridnames{14}{3}='r6A';
gridnames{14}{4}='r6R'; gridnames{14}{5}='';
%gridnames{15}{1}='r7F'; gridnames{15}{2}='r7B'; gridnames{15}{3}='r7A';
gridnames{15}{4}='r7R'; gridnames{15}{5}='';
%gridnames{16}{1}='r8F'; gridnames{16}{2}='r8B'; gridnames{16}{3}='r8A';
gridnames{16}{4}='r8R'; gridnames{16}{5}='';
%gridnames{17}{1}='r9F'; gridnames{17}{2}='r9B'; gridnames{17}{3}='r9A';
gridnames{17}{4}='r9R'; gridnames{17}{5}='';
%gridnames{18}{1}='r10F'; gridnames{18}{2}='r10B';
gridnames{18}{3}='r10A'; gridnames{18}{4}='r10R'; gridnames{18}{5}='';
%gridnames{19}{1}='r11F'; gridnames{19}{2}='r11B';
gridnames{19}{3}='r11A'; gridnames{19}{4}='r11R'; gridnames{19}{5}='';
%gridnames{20}{1}='my1F'; gridnames{20}{2}='my1B';
gridnames{20}{3}='my1A'; gridnames{20}{4}='my1R'; gridnames{20}{5}='';
%times{1}='E11.5';
%times{2}='E13.5';
%times{3}='E15.5';
%times{4}='E18.5';
%times{5}='P4';
%times{6}='P14';
%times{7}='P28';


function ABAcodes_mainprogram(ngenes, nx, ny, gridnames, ntimesteps,
times, braindata, outputfilename)


%%%%%%%%%%%%%%%%%%%%%%%%%%%%%%%%%%%%%%%%%%%%%%%%%%%%%%%%%%%%%%%%%%%%%%%%%%%%
%%%
% Generate robust components for each gene expression data.
%%%%%%%%%%%%%%%%%%%%%%%%%%%%%%%%%%%%%%%%%%%%%%%%%%%%%%%%%%%%%%%%%%%%%%%%%%%%
%%%

% Vary thresholds of expression values and choose the clustering with the
longest durations in threshold values.
lwdvalthre=0.2;
uppvalthre=1.0;
tmp=braindata(:);
tmp(find(~(tmp>=0)))=0;
tmp=tmp(find(tmp>=0));
valthre=prctile(tmp,70);
clear genecomponents;
xlabels=zeros(nx,ny);
for i1=1:nx
 for i2=1:ny
  str=gridnames{i1}{i2};
  if (length(str)>0)
   xlabels(i1,i2)=1;
  end
 end
end

for n=1:ngenes
```

```
 for t=1:ntimesteps
  twodmat=zeros(nx,ny);
  for i1=1:nx
   for i2=1:ny
    twodmat(i1,i2)=braindata(n,i1,i2,t);
   end
  end

[ncomponents,components]=detect_stable_clusters(twodmat,xlabels,lwdvalthre
,uppvalthre,valthre);
  genecomponents{n}{t}.ncomponents=ncomponents;
  genecomponents{n}{t}.components=components;
 end

end



%%%%%%%%%%%%%%%%%%%%%%%%%%%%%%%%%%%%%%%%%%%%%%%%%%%%%%%%%%%%%%%%%%%%%%%%%%
%%%
% Determine whole-brain expression and no expression patterns.
%%%%%%%%%%%%%%%%%%%%%%%%%%%%%%%%%%%%%%%%%%%%%%%%%%%%%%%%%%%%%%%%%%%%%%%%%%
%%%

% If the marked components exceed half of the substructures with valid
data, then label it as whole-brain expressed.
expressionstates=zeros(ngenes,ntimesteps);
for n=1:ngenes
 for t=1:ntimesteps
  twodmat=zeros(nx,ny);
  for i1=1:nx
   for i2=1:ny
    twodmat(i1,i2)=braindata(n,i1,i2,t);
   end
  end
  zz=zeros(nx,ny);
  for i=1:genecomponents{n}{t}.ncomponents
   cs=genecomponents{n}{t}.components{i};
   for j=1:length(cs(:,1))
    zz(cs(j,1),cs(j,2))=1;
   end
  end
  n1=sum(sum(twodmat>=0)); n2=sum(sum((zz==1)&(twodmat>=0)));
  if ((n1>0)&(n2>=(0.5*n1)))
   expressionstates(n,t)=1;
  end
 end
end



%%%%%%%%%%%%%%%%%%%%%%%%%%%%%%%%%%%%%%%%%%%%%%%%%%%%%%%%%%%%%%%%%%%%%%%%%%
%%%%
```

```
% Clean up components.
%%%%%%%%%%%%%%%%%%%%%%%%%%%%%%%%%%%%%%%%%%%%%%%%%%%%%%%%%%%%%%%%%%%%%%%%%%%%%%
%%%%

% Obtain the distributions of top k clust sizes by randomly labeling
substructures on 2D grids.
% Pr(substructure=1) depends on the expression value threshold.
valthres=zeros(1,6); tmp=braindata(:); tmp=tmp(find(tmp>=0));
ndraws=10000; ntops=10;
clear clsizedists;
for i=1:6
 valthres(i)=prctile(tmp,(i+3)*10);
 val=valthres(i);

clsizedists(:,:,i)=find_random_cluster_size_distribution(braindata,xlabels
,val,ndraws,ntops);
end


% Keep the components with significant sizes.
% Use background random cluster size distribution to determine the cluster
size thresholds.
% If the component sizes do not pass the threshold values, then check
whether they appear multiple times in the time series.
bkgddist=clsizedists(:,:,4); pvalthre=0.1;
clear genecompsizepvals genecompselected;
for n=1:ngenes
 for t=1:ntimesteps
  twodmat=zeros(nx,ny);
  for i1=1:nx
   for i2=1:ny
    twodmat(i1,i2)=braindata(n,i1,i2,t);
   end
  end
  genecompselected{n}{t}=[]; flag=1;
  for i=1:genecomponents{n}{t}.ncomponents
   subset=genecomponents{n}{t}.components{i};
   k=0;
   for l=1:length(subset(:,1))
    if (twodmat(subset(l,1),subset(l,2))>=0)
     k=k+1;
    end
   end
   j=k+1;
   val=sum(bkgddist(i,1:j))/ndraws; val=1-val;
   genecompsizepvals{n}{t}(i)=val;
   if ((val<=pvalthre)&(flag==1))
    genecompselected{n}{t}(i)=1;
   else
    genecompselected{n}{t}(i)=0;
    flag=0;
   end
  end
 end
end
```

```
% Reactivate small but recurrent components.
% The matched components at other time steps should not be too large.
% Very small components (<=2 substructures) have to find matched ones in
two more time points.
% Discard very small components (<=2 substructures).
distthre=2; newgenecompselected=genecompselected; foldthre=3;
smallcompsize=2;
for n=1:ngenes
 for t1=1:ntimesteps
  for i1=1:genecomponents{n}{t1}.ncomponents
   if (genecompselected{n}{t1}(i1)==0)
    matches=zeros(1,ntimesteps);
    for t2=1:ntimesteps
     if (t1~=t2)
      for i2=1:genecomponents{n}{t2}.ncomponents
       points1=genecomponents{n}{t1}.components{i1};
       points2=genecomponents{n}{t2}.components{i2};
       n1=length(points1(:,1)); n2=length(points2(:,1));
       dists=nx*ny*ones(n1,n2);
       for j1=1:n1
      for j2=1:n2
         d=points1(j1,:)-points2(j2,:); dists(j1,j2)=norm(d,2);
        end
       end
       if
((min(min(dists(:)))<=distthre)&(n1<=(foldthre*n2))&(n2<=(foldthre*n1)))
      matches(t2)=matches(t2)+1;
       end
      end
     end
    end
    if (n1<=smallcompsize)
     newgenecompselected{n}{t1}(i1)=0;
    elseif ((n1>smallcompsize)&(sum(matches>0)>0))
     newgenecompselected{n}{t1}(i1)=1;
    end
   end
  end
 end
end


genecompselected=newgenecompselected;


% Remove the discarded components.
clear newgenecomponents;
for n=1:ngenes
 for t=1:ntimesteps
  twodmat=zeros(nx,ny);
  for i1=1:nx
   for i2=1:ny
    twodmat(i1,i2)=braindata(n,i1,i2,t);
```

```
   end
  end
  subset=find(genecompselected{n}{t}==1);
  if (length(subset)<=0)
   newgenecomponents{n}{t}.ncomponents=0;
   newgenecomponents{n}{t}.components={};
  else
   newgenecomponents{n}{t}.ncomponents=length(subset);
   for i=1:newgenecomponents{n}{t}.ncomponents
    j=subset(i);
    points=genecomponents{n}{t}.components{j};
    selected=zeros(1,length(points(:,1)));
    for k=1:length(selected)
     if (twodmat(points(k,1),points(k,2))>=0)
       selected(k)=1;
     end
    end
    points=points(find(selected==1),:);
    newgenecomponents{n}{t}.components{i}=points;
   end
  end
 end
end


% Generate mappings between components.
distthre=2; foldthre=2; comparablefoldthre=0.75; smallcompsizethre=2;
largecompsizethre=20;
clear genelineages ngenetotalcomps genetotalcomplists genecompmappings
genemappingtypes genelineagetopologies;
for n=1:ngenes

[ngenetotalcomps{n},genetotalcomplists{n},genecompmappings{n}]=generate_co
mpmapping(braindata,newgenecomponents{n},expressionstates(n,:),distthre,fo
ldthre,comparablefoldthre,smallcompsizethre,largecompsizethre);
end




%%%%%%%%%%%%%%%%%%%%%%%%%%%%%%%%%%%%%%%%%%%%%%%%%%%%%%%%%%%%%%%%%%%%%%%%%%
%%%%
% Find localized spatial-temporal expression patterns.
%%%%%%%%%%%%%%%%%%%%%%%%%%%%%%%%%%%%%%%%%%%%%%%%%%%%%%%%%%%%%%%%%%%%%%%%%%
%%%%

% A component is localized if (1)its conex hull is not much larger than
the component, (2)it is not very large.
% Only consider the components with lineage relations to other components.
largecompthre=50; fracthre=0.75; holethre=8; largecompfracthre=0.5;
clear localized;
for n=1:ngenes
 for t=1:ntimesteps
  localized{n}{t}=zeros(1,newgenecomponents{n}{t}.ncomponents);
```

```
  if (expressionstates(n,t)<=0)
   twodmat=zeros(nx,ny);
   for i1=1:nx
    for i2=1:ny
     twodmat(i1,i2)=braindata(n,i1,i2,t);
    end
   end
   nvalidentries=sum(twodmat(:)>=0);
   for i=1:newgenecomponents{n}{t}.ncomponents

ind=find((genetotalcomplists{n}(:,1)==t)&(genetotalcomplists{n}(:,2)==i));
    if
((sum(genecompmappings{n}(ind,:)>0)>0)|(sum(genecompmappings{n}(:,ind)>0)>
0))
     points=newgenecomponents{n}{t}.components{i};
npoints=length(points(:,1));
     s=find((points(:,1)>=0)&(points(:,2)>=0));
     if (((length(s)/nvalidentries)<=largecompfracthre)&(npoints>2))
      % Handle the colinear cases.
      if (collinear(points)==1)
       if (length(unique(points(:,1)))>1)
        [yy,ii]=sort(points(:,1)); points=points(ii,:);
K=transpose([1:npoints 1]);
       else
        [yy,ii]=sort(points(:,2)); points=points(ii,:);
K=transpose([1:npoints 1]);
       end
      else
       K=convhull(points(:,1),points(:,2));
      end
      minx=min(points(:,1)); maxx=max(points(:,1));
      miny=min(points(:,2)); maxy=max(points(:,2));
      ncands=0; cands=[];
      for x=minx:maxx
       for y=miny:maxy
        if (twodmat(x,y)>=0)
         ncands=ncands+1; cands(ncands,:)=[x y];
        end
       end
      end
      indicators=inpolygon(cands(:,1),cands(:,2),points(K,1),points(K,2));
      r=npoints/sum(indicators==1);
      if ((r>=fracthre)&((sum(indicators==1)-npoints)<holethre))
       localized{n}{t}(i)=1;
      end
     end
    end
   end
  end
 end
end



% Extract localized clusters.
```

```
nlocalclusters=0; clear localclusters localclusterinfo;
for n=1:ngenes
 for t=1:ntimesteps
  for i=1:newgenecomponents{n}{t}.ncomponents;
   if
((localized{n}{t}(i)==1)&(length(newgenecomponents{n}{t}.components{i}(:,1
))>=5))
    nlocalclusters=nlocalclusters+1;
    localclusterinfo(nlocalclusters,:)=[n t i];
    localclusters{nlocalclusters}=newgenecomponents{n}{t}.components{i};
   end
  end
 end
end


% Group localized clusters together.
% Compute pairwise similarities of clusters.
% Similarity of clusters i and j are |intersect(i,j)|/max(|cluster
i|,|cluster j|).
clusim=zeros(nlocalclusters,nlocalclusters);
for n=1:(nlocalclusters-1)
 clusim(n,n)=1;
 zz1=zeros(nx,ny);
 for i=1:length(localclusters{n}(:,1))

zz1(localclusters{n}(i,1),localclusters{n}(i,2))=zz1(localclusters{n}(i,1)
,localclusters{n}(i,2))+1;
 end
 k1=sum(sum(zz1>0));
 for m=(n+1):nlocalclusters
  zz2=zeros(nx,ny);
  for i=1:length(localclusters{m}(:,1))

zz2(localclusters{m}(i,1),localclusters{m}(i,2))=zz2(localclusters{m}(i,1)
,localclusters{m}(i,2))+1;
  end
  k2=sum(sum(zz2>0));
  k3=sum(sum((zz1>0)&(zz2>0)));
  clusim(n,m)=k3/max(k1,k2);
  clusim(m,n)=clusim(n,m);
 end
end


% Find cliques and clusters from the similarity graph.
wthre1=0.5; wthre2=0.4;

[ncliques,cliques,nclus,clus]=find_cliques_clusters(clusim,wthre1,wthre2);


% Groups consist of >= 10 localized clusters.
ngroups=0; clear groups; sizethre=10;
for n=1:nclus
 if (length(clus{n})>=sizethre)
```

```
  ngroups=ngroups+1; groups{ngroups}=clus{n};
 end
end


% Assign genes to each group.
% One gene may consists of patterns belonging to multiple groups.
% Also, specify assignments for each time point.
groupassignments=zeros(ntimesteps,ngroups,ngenes);
for m=1:ngroups
 for n=1:length(groups{m})
  i=groups{m}(n); ind=localclusterinfo(i,1); t=localclusterinfo(i,2);
  groupassignments(t,m,ind)=1;
 end
end


save(outputfilename,'genecomponents','expressionstates','newgenecomponents
','nlocalclusters','localclusters','groupassignments');






%%%%%%%%%%%%%%%%%%%%%%%%%%%%%%%%%%%%%%%%%%%%%%%%%%%%%%%%%%%%%%%%%%%%%%%%%%%
% detect_stable_clusters.m
%%%%%%%%%%%%%%%%%%%%%%%%%%%%%%%%%%%%%%%%%%%%%%%%%%%%%%%%%%%%%%%%%%%%%%%%%%%

% Detect stable clusters on 2D grids.
% Vary the threshold value and trace the evolution of each connected
component.  Report components with long survival durations.
% If the upper limit of the first interval is small, then do not choose
the first interval as many weak points will be included.

function [ncomponents, components] = detect_stable_clusters(twodmat,
refmat, lwdvalthre, uppvalthre, valthre)


[nx,ny]=size(twodmat);


vals=twodmat(:); vals=unique(vals);
vals=vals(find((vals>=0)|(vals<0)));
vals=sort(vals);


% Fill the missing entries with the neighboring valid values.
newtwodmat=twodmat;
for i1=1:nx
 for i2=1:ny
  if ((refmat(i1,i2)>0)&(~(twodmat(i1,i2)>=0)))
   nentries=0; entries=[];
   if ((i1>1)&(twodmat(i1-1,i2)>=0))
    nentries=nentries+1; entries(nentries)=twodmat(i1-1,i2);
```

```
   elseif ((i1<nx)&(twodmat(i1+1,i2)>=0))
    nentries=nentries+1; entries(nentries)=twodmat(i1+1,i2);
   elseif ((i2>1)&(twodmat(i1,i2-1)>=0))
    nentries=nentries+1; entries(nentries)=twodmat(i1,i2-1);
   elseif ((i2<ny)&(twodmat(i1,i2+1)>=0))
    nentries=nentries+1; entries(nentries)=twodmat(i1,i2+1);
   elseif ((i1>1)&(i2>1)&(twodmat(i1-1,i2-1)>=0))
    nentries=nentries+1; entries(nentries)=twodmat(i1-1,i2-1);
   elseif ((i1<nx)&(i2>1)&(twodmat(i1+1,i2-1)>=0))
    nentries=nentries+1; entries(nentries)=twodmat(i1+1,i2-1);
   elseif ((i1>1)&(i2<ny)&(twodmat(i1-1,i2+1)>=0))
    nentries=nentries+1; entries(nentries)=twodmat(i1-1,i2+1);
   elseif ((i1<nx)&(i2<ny)&(twodmat(i1+1,i2+1)>=0))
    nentries=nentries+1; entries(nentries)=twodmat(i1+1,i2+1);
   end
   newtwodmat(i1,i2)=mean(entries);
  end
 end
end



nnodes=0; coords=[];
for i1=1:nx
 for i2=1:ny
  if (refmat(i1,i2)>0)
   nnodes=nnodes+1; coords(nnodes,:)=[i1 i2];
  end
 end
end


% If the minimum value >= uppvalthre, then all data points belong to one
component.
if (vals(1)>=uppvalthre)
 ncomponents=1;
 components{1}=coords;
 return;
end

% If the maximum value <= lwdvalthre, then there is no component.
if (vals(length(vals))<=lwdvalthre)
 ncomponents=0; components=[];
 return;
end


G=zeros(nnodes,nnodes);
for i=1:nnodes
 c1=coords(i,1:2); G(i,i)=1;
 for j=(i+1):nnodes
  c2=coords(j,1:2); flag=0;
  if ((c1(1)==c2(1))&(abs(c1(2)-c2(2))==1))
   flag=1;
  elseif ((c1(2)==c2(2))&(abs(c1(1)-c2(1))==1))
```

```
   flag=1;
  elseif ((abs(c1(1)-c2(1))==1)&(abs(c1(2)-c2(2))==1))
   flag=1;
  end
  if (flag==1)
   G(i,j)=1; G(j,i)=1;
  end
 end
end



% Generate the evolutionary history of clusters.
% Record the number of cluster for each threshold value.
nclusters=0; clusters={}; ncs=zeros(1,length(vals));
for ind=1:length(vals)
 val=vals(ind);
 [a,b]=find(newtwodmat>=val);
 nsubnodes=length(a);
 inds=zeros(1,nsubnodes);
 for i=1:nsubnodes
  k=find((coords(:,1)==a(i))&(coords(:,2)==b(i)));
  inds(i)=k;
 end
 subG=G(inds,inds);
 [ncomps,comps]=find_conn_comps(nsubnodes,subG);
 ncs(ind)=ncomps;
 nnewclusters=nclusters; newclusters=clusters;
 for n=1:ncomps
  pas=[]; npas=0;
  for i=1:nclusters
   if (sum(ismember(comps{n}.comps,clusters{i}.comps)==1)==comps{n}.n)
    npas=npas+1; pas(npas,:)=[i clusters{i}.birth];
   end
  end
  if (npas>0)
   k=find(pas(:,2)>=max(pas(:,2))); k=pas(k,1);
  else
   k=0;
  end
  nnewclusters=nnewclusters+1;
  newclusters{nnewclusters}.birth=ind;
  newclusters{nnewclusters}.death=length(vals);
  newclusters{nnewclusters}.pa=k;
  newclusters{nnewclusters}.chl=[];
  if (k>0)
   newclusters{k}.death=ind;
   newclusters{k}.chl(1+length(newclusters{k}.chl))=nnewclusters;
  end
  newclusters{nnewclusters}.comps=comps{n}.comps;
 end
 nclusters=nnewclusters; clusters=newclusters;
end
```

```
% Calculate the survival duration of each threshold interval.
nintervals=1; intervals=[1 0]; curn=ncs(1);
for ind=2:length(vals)
 if (ncs(ind)~=curn)
  intervals(nintervals,2)=ind-1; curn=ncs(ind);
  nintervals=nintervals+1; intervals(nintervals,1)=ind;
 end
end
intervals(nintervals,2)=length(vals);
intervalds=intervals(:,2)-intervals(:,1);


% Only consider the intervals within the range of [lwdvalthre,uppvalthre].
% If the upper value of the first interval < valthre, then do not consider
the first interval.

isselected=zeros(1,nintervals);
startind=min(find(vals>=lwdvalthre));
endind=max(find(vals<=uppvalthre));
for i=1:nintervals
 if ((intervals(i,2)>=startind)&(intervals(i,1)<=endind))
  isselected(i)=1;
 end
end
if ((nintervals>1)&(vals(intervals(1,2))<valthre))
 isselected(1)=0;
end
selected=find(isselected==1);
if (length(selected)>0)
 intervalds=intervals(selected,2)-intervals(selected,1);
 k=find(intervalds>=max(intervalds)); k=k(1); k=selected(k);
 val1=vals(intervals(k,1));
 val2=vals(intervals(k,2));
 if (val1<lwdvalthre)
  if (val2>uppvalthre)
   %val=lwdvalthre;
   val=valthre;
  else
   val=val2;
  end
 else
  val=val1;
 end
else
 val=valthre;
end



[a,b]=find(newtwodmat>=val);
nsubnodes=length(a);
inds=zeros(1,nsubnodes);
for i=1:nsubnodes
 k=find((coords(:,1)==a(i))&(coords(:,2)==b(i)));
 inds(i)=k;
```

```
end
subG=G(inds,inds);
[ncomps,comps]=find_conn_comps(nsubnodes,subG);
ncomponents=ncomps; clear components;
for n=1:ncomponents
 components{n}=zeros(comps{n}.n,2);
 for i=1:comps{n}.n
  j=inds(comps{n}.comps(i));
  components{n}(i,:)=[coords(j,1) coords(j,2)];
 end
end




%%%%%%%%%%%%%%%%%%%%%%%%%%%%%%%%%%%%%%%%%%%%%%%%%%%%%%%%%%%%%%%%%%%%%%%
% find_random_cluster_size_distribution.m.
%%%%%%%%%%%%%%%%%%%%%%%%%%%%%%%%%%%%%%%%%%%%%%%%%%%%%%%%%%%%%%%%%%%%%%%

% Calculate the distribution of top k cluster sizes when randomly set
substructures in a 2D grid to 1s.
% The probability of setting value 1 is the probability that the
substructure expression >= the given threshold value.
% Use this distribution to determine the small clusters to throw away when
constructing the lineages of spatial-temporal patterns.

function clsizedists = find_random_cluster_size_distribution(bkgdata,
refmat, valthre, ndraws, ntops)


% Determine the probability of setting value 1.
tmp=bkgdata(:); tmp=tmp(find(tmp>=0)); p=sum(tmp>=valthre)/length(tmp);
[nx,ny]=size(refmat);


nnodes=0; coords=[];
for i1=1:nx
 for i2=1:ny
  if (refmat(i1,i2)>0)
   nnodes=nnodes+1; coords(nnodes,:)=[i1 i2];
  end
 end
end

G=zeros(nnodes,nnodes);
for i=1:nnodes
 c1=coords(i,1:2); G(i,i)=1;
 for j=(i+1):nnodes
  c2=coords(j,1:2); flag=0;
  if ((c1(1)==c2(1))&(abs(c1(2)-c2(2))==1))
   flag=1;
  elseif ((c1(2)==c2(2))&(abs(c1(1)-c2(1))==1))
   flag=1;
  elseif ((abs(c1(1)-c2(1))==1)&(abs(c1(2)-c2(2))==1))
   flag=1;
```

```
   end
   if (flag==1)
    G(i,j)=1; G(j,i)=1;
   end
  end
 end


% Randomly generate binary patterns and count the max cluster size.
maxclsize=sum(refmat(:)>0); clsizedists=zeros(ntops,maxclsize+1);
for drawind=1:ndraws

 randmat=rand(nx,ny); randmat(find(refmat<=0))=0;
 tmpmat=zeros(nx,ny);
 tmpmat(find(randmat>=(1-p)))=1;
 [a,b]=find(tmpmat>0);
 nsubnodes=length(a);
 inds=zeros(1,nsubnodes);
 for i=1:nsubnodes
  k=find((coords(:,1)==a(i))&(coords(:,2)==b(i)));
  inds(i)=k;
 end

 if (length(inds)<=0)
  ncomps=0;

 else
  subG=G(inds,inds);
  [ncomps,comps]=find_conn_comps(nsubnodes,subG);
 end


 % Count the top k cluster sizes.
 % The components obtained from find_conn_comps are sorted by their sizes.
 for k=1:ntops
  if (k<=ncomps)
   l=comps{k}.n;
  else
   l=0;
  end
  clsizedists(k,l+1)=clsizedists(k,l+1)+1;
 end


end



%%%%%%%%%%%%%%%%%%%%%%%%%%%%%%%%%%%%%%%%%%%%%%%%%%%%%%%%%%%%%%%%%%%%%%%%%%%%%%%%
% generate_compmapping.m.
%%%%%%%%%%%%%%%%%%%%%%%%%%%%%%%%%%%%%%%%%%%%%%%%%%%%%%%%%%%%%%%%%%%%%%%%%%%%%%%%

% Generate the mappings between components.
```

```
function [ntotalcomps, totalcomplist, compmappings] =
generate_compmapping(genedata, genecomponent, expressionstate, distthre,
foldthre, comparablefoldthre, smallcompsizethre, largecompsizethre)


[subgenes,nx,ny,ntimesteps]=size(genedata);


% Establish possible links between components.
% Two components are linked if they satisfy the following conditions:
% (1)Component sizes > smallcompsizethre.  (2)One precedes the other.
(3)Their locations are overlapped or close.  (4)None of them specifies
whole-brain expression.
nnodes=0; nodes=[];
for t=1:ntimesteps
  for i=1:genecomponent{t}.ncomponents
   nnodes=nnodes+1; nodes(nnodes,:)=[t i];
 end
end
G=zeros(nnodes,nnodes);
for ind1=1:nnodes
 for ind2=1:nnodes
  if ((ind1~=ind2)&(nodes(ind1,1)<nodes(ind2,1)))
   t1=nodes(ind1,1); i1=nodes(ind1,2);
   t2=nodes(ind2,1); i2=nodes(ind2,2);
   n1=length(genecomponent{t1}.components{i1}(:,1));
   n2=length(genecomponent{t2}.components{i2}(:,1));
   if
((expressionstate(t1)~=1)&(expressionstate(t2)~=1)&(n1>smallcompsizethre)&
(n2>smallcompsizethre))
    points1=genecomponent{t1}.components{i1};
    points2=genecomponent{t2}.components{i2};
    d=mindist(points1,points2);
    if (d<=distthre)
      G(ind1,ind2)=1;
    end
   end
  end
 end
end



% Filter out noisy links.
% (1)If one component links to components at multiple time points, then
only keep the links with the closest time point.
newG=zeros(nnodes,nnodes);
for ind=1:nnodes
 subset=find(G(ind,:)>0);
 if (length(subset)>0)
  ts=nodes(subset,1); mint=min(ts);
  subset2=subset(find(ts<=mint));
  newG(ind,subset2)=1;
 end
end
```

```
% (2)If one component links to multiple components, then either only one
heritage link sustains or this is a fission event.
% If only one child component has a comparable size to the parent
component and all other child components are small, then discard the links
to small components.
% If no child component has a comparable size to the parent component and
all the child components are smaller than the parent component, then keep
the links to all components.
% If multiple child components have comparable or larger sizes as the
parent component, then only keep one link to the component of the most
similar size.
% If multiple child components have comparable or larger sizes as the
parent component, then only keep one links to the component of the closest
distance.
G=newG;
newG=zeros(nnodes,nnodes);
for ind=1:nnodes
 subset=find(G(ind,:)>0);
 if (length(subset)>1)
  cpa=length(genecomponent{nodes(ind,1)}.components{nodes(ind,2)}(:,1));
  cchildren=zeros(1,length(subset));
  for i=1:length(subset)

cchildren(i)=length(genecomponent{nodes(subset(i),1)}.components{nodes(sub
set(i),2)}(:,1));
  end
  nlarge=sum(cchildren>=(comparablefoldthre*cpa));
  nsmall=sum(cchildren<(comparablefoldthre*cpa));
  if (nlarge==1)
   k=find(cchildren>=(comparablefoldthre*cpa)); k=subset(k);
   newG(ind,k)=1;
  elseif (nlarge==0)
   newG(ind,subset)=1;
  else
   ds=1e10*ones(1,length(cchildren));
   for i=1:length(subset)
    points1=genecomponent{nodes(ind,1)}.components{nodes(ind,2)};

points2=genecomponent{nodes(subset(i),1)}.components{nodes(subset(i),2)};
    ds(i)=mindist(points1,points2);
   end
   k=find(ds<=min(ds)); k=subset(k);

   %cr=abs(log2(cchildren/cpa));
   %k=find(cr<=min(cr)); k=subset(k);
   %k=find(cchildren>=max(cchildren)); k=subset(k);
   newG(ind,k)=1;
  end
 elseif (length(subset)==1)
  newG(ind,subset)=1;
 end
end
```

```
% (3)If multiple components link to one component, then either only one
heritage link sustains or this is a fusion event.
% If only one parent component has a comparable size to the child
component and all other parent components are small, then discard the
links from small components.
% If no parent component has a comparable size to the child component and
all the parent components are smaller than the child component, then keep
the links from all components.
% If multiple parent components have comparable or larger sizes as the
child component, then only keep one link from the component of the most
similar size.
% If multiple parent components have comparable or larger sizes as the
child component, then only keep one link from the component of the closest
distance.
G=newG;
newG=zeros(nnodes,nnodes);
for ind=1:nnodes
 subset=find(G(:,ind)>0);
 if (length(subset)>1)

cchild=length(genecomponent{nodes(ind,1)}.components{nodes(ind,2)}(:,1));
  cpas=zeros(1,length(subset));
  for i=1:length(subset)

cpas(i)=length(genecomponent{nodes(subset(i),1)}.components{nodes(subset(i
),2)}(:,1));
  end
  nlarge=sum(cpas>=(comparablefoldthre*cchild));
  nsmall=sum(cpas<(comparablefoldthre*cchild));
  if (nlarge==1)
   k=find(cpas>=(comparablefoldthre*cchild)); k=subset(k);
   newG(k,ind)=1;
  elseif (nlarge==0)
   newG(subset,ind)=1;
  else
   ds=1e10*ones(1,length(cpas));
   for i=1:length(subset)
    points1=genecomponent{nodes(ind,1)}.components{nodes(ind,2)};

points2=genecomponent{nodes(subset(i),1)}.components{nodes(subset(i),2)};
    ds(i)=mindist(points1,points2);
   end
   k=find(ds<=min(ds)); k=subset(k);

   %cr=abs(log2(cpas/cchild));
   %k=find(cr<=min(cr)); k=subset(k);
   %k=find(cpas>=max(cpas)); k=subset(k);
   newG(k,ind)=1;
  end
 elseif (length(subset)==1)
  newG(subset,ind)=1;
 end
end
G=newG;
```

```
ntotalcomps=nnodes;
totalcomplist=nodes;
compmappings=G;



%%%%%%%%%%%%%%%%%%%%%%%%%%%%%%%%%%%%%%%%%%%%%%%%%%%%%%%%%%%%%%%%%%%%%%%%%%%%
% mindist.m.
%%%%%%%%%%%%%%%%%%%%%%%%%%%%%%%%%%%%%%%%%%%%%%%%%%%%%%%%%%%%%%%%%%%%%%%%%%%%

% Calculate the minimum distance between two sets of points.
% Discard the points with NaN entries.

function md = mindist(points1, points2)

n1=length(points1(:,1)); n2=length(points2(:,1));
dists=1e20*ones(n1,n2);
for i=1:n1
 for j=1:n2
  vec1=points1(i,:); vec2=points2(j,:);
  if ((sum(~((vec1>=0)|(vec1<=0)))==0)&(sum(~((vec2>=0)|(vec2<=0)))==0))
   d=vec1-vec2; dists(i,j)=norm(d,2);
  end
 end
end

md=min(dists(:));




%%%%%%%%%%%%%%%%%%%%%%%%%%%%%%%%%%%%%%%%%%%%%%%%%%%%%%%%%%%%%%%%%%%%%%%%%%%%
% find_cliques_clusters.m.
%%%%%%%%%%%%%%%%%%%%%%%%%%%%%%%%%%%%%%%%%%%%%%%%%%%%%%%%%%%%%%%%%%%%%%%%%%%%

% Find maximal cliques and clusters in an undirected graph.
% wthre1=0.8; wthre2=0.65;

function [ncliques, cliques, nclus, clus] = find_cliques_clusters(W,
wthre1, wthre2)

[nnodes,nnodes]=size(W);

% Sort graph similarities above the threshold.
nweights=0; clear weights;
[a,b]=find(W>=wthre1);
ss=find(a<b);
weights(:,1)=a(ss); weights(:,2)=b(ss);
nweights=length(weights(:,1));
for i=1:nweights
 weights(i,3)=W(weights(i,1),weights(i,2));
end

%nweights=0; clear weights;
%for n=1:nnodes
```

```
% for m=(n+1):nnodes
%  if (W(n,m)>=wthre1)
%   nweights=nweights+1; weights(nweights,:)=[n m W(n,m)];
%  end
% end
%end

[Y,I]=sort(unique(weights(:,3)),'descend'); sweights=Y;
nsweights=length(sweights);

ncliques=0; clear cliques; flag=1; curind=1; thre=sweights(curind);


while (flag==1)

 % Try to merge cliques obtained from previous steps with the new edges.
 clmemberships=zeros(1,nnodes);
 if (ncliques>0)
  G=zeros(ncliques,ncliques);
  for i=1:ncliques
   set1=cliques{i}; G(i,i)=1;
   for j=(i+1):ncliques
    set2=cliques{j};
    if (sum(sum(W(set1,set2)<thre))==0)
     G(i,j)=1; G(j,i)=1;
    end
   end
  end
  [ncomps,comps]=find_conn_comps(ncliques,G);
  newids=zeros(1,ncliques); nnewids=0;
  for n=1:ncomps
   subset=comps{n}.comps;
   if (sum(sum(G(subset,subset)==0))==0)
    nnewids=nnewids+1; newids(subset)=nnewids;
   else
    tmpG=G(subset,subset);
    visited=zeros(1,length(subset)); flag2=1;
    while (flag2==1)
     subset2=find(visited==0);
     if (length(subset2)==0)
      flag2=0;
     else
      ds=sum(tmpG(:,subset2));
      vs=zeros(1,length(subset2));
      for i=1:length(subset2)
       subset3=[find(visited==1) subset2(i)];
       if (sum(sum(tmpG(subset3,subset3)==0))==0)
        vs(i)=1;
       end
      end
      if (sum(vs==1)>0)
       maxd=max(ds(find(vs==1)));
       i=find((ds>=maxd)&(vs==1)); i=i(1);
       visited(subset2(i))=1;
      else
```

```
      flag2=0;
     end
    end
   end
   tmpset=subset(find(visited==1));
   nnewids=nnewids+1; newids(tmpset)=nnewids;
   for i=1:length(subset)
    j=subset(i);
    if (ismember(j,tmpset)==0)
      nnewids=nnewids+1; newids(j)=nnewids;
    end
   end
  end
 end
 for n=1:ncliques
  k=newids(n); clmemberships(cliques{n})=k;
 end

 nnewcliques=nnewids; clear newcliques;
 for n=1:nnewcliques
  newcliques{n}=find(clmemberships==n);
 end
 ncliques=nnewcliques; cliques=newcliques;

end


% Sort nodes not belonging to existing cliques according to their
connectivity.
conns=-1*ones(1,nnodes);
for n=1:nnodes
 if (clmemberships(n)<=0)
  k=sum(W(n,:)>=thre); conns(n)=k;
 end
end
[sortedconns,sortednodes]=sort(conns,'descend');


% Try to assign newly emerged nodes to existing cliques.
% If there are multiple candidate cliques then choose the largest one.
if (ncliques>0)
 for m=1:nnodes
  if (sortedconns(m)>0)
   n=sortednodes(m); cands=[]; candsizes=[];
   for i=1:ncliques
    if (sum(W(n,cliques{i})<thre)==0)
     cands(1+length(cands))=i;
     candsizes(1+length(candsizes))=length(cliques{i});
    end
   end
   if (length(cands)>0)
    [Y,I]=sort(candsizes,'descend'); k=cands(I(1));
    clmemberships(n)=k;
   end
  end
```

```
 end
 for n=1:ncliques
  subset=find(clmemberships==n);
  cliques{n}=subset;
 end
end


% Try to generate new cliques from the newly emerged nodes.
% Start with singleton cliques.
conns=zeros(1,nnodes);
subset=find(clmemberships<=0);
for n=1:nnodes
 if (clmemberships(n)<=0)
  conns(n)=sum(W(n,subset)>=thre);
 end
end
[Y,I]=sort(conns,'descend');
nnewcliques=sum(conns>0); clear newcliques;
for n=1:nnewcliques
 newcliques{n}=I(n);
end


% Iteratively merge two cliques until no pairs are mergeable.
flag2=1;
while (flag2==1)
 mergeable=0; i=1; inds=zeros(1,2);
 while ((i<=(nnewcliques-1))&(mergeable==0))
  j=i+1; set1=newcliques{i};
  while ((j<=nnewcliques)&(mergeable==0))
   set2=newcliques{j};
   if (sum(sum(W(set1,set2)<thre))==0)
    mergeable=1; inds=[i j];
   end
   j=j+1;
  end
  i=i+1;
 end
 if (mergeable==1)
  i=inds(1); j=inds(2);
  newcliques{i}=union(newcliques{i},newcliques{j});
  for k=j:(nnewcliques-1)
   newcliques{k}=newcliques{k+1};
  end
  nnewcliques=nnewcliques-1;
 else
  flag2=0;
 end
end

% Add new cliques to the records.
% Only consider cliques with more than one member.
n=1;
%for n=1:nnewcliques
```

```
 while ((n<=nnewcliques)&(length(newcliques{n})>1))
  %if (length(newcliques{n})>1)
  cliques{ncliques+n}=newcliques{n};
  %end
  n=n+1;
 end
 %ncliques=nnewcliques;
 ncliques=ncliques+n-1;


 % Sort cliques by their sizes.
 clsizes=zeros(1,ncliques);
 for n=1:ncliques
  clsizes(n)=length(cliques{n});
 end
 [Y,I]=sort(clsizes,'descend');
 clear newcliques nnewcliques;
 for n=1:ncliques
  newcliques{n}=cliques{I(n)};
 end
 cliques=newcliques;

 % Stop when curind reaches the upper limit.
 curind=curind+1;
 if (curind>nsweights)
  flag=0;
 else
  thre=sweights(curind);
  if (thre<wthre1)
   flag=0;
  end
 end

end



% Merge cliques into clusters.
nclus=ncliques; clus=cliques; flag=1;

while (flag==1)

 % Sort clusters by sizes.
 clsizes=zeros(1,nclus);
 for n=1:nclus
  clsizes(n)=length(clus{n});
 end
 [Y,I]=sort(clsizes,'descend');
 clear newclus;
 for m=1:nclus
  n=I(m); newclus{m}=clus{n};
 end
 clus=newclus; clear newclus;

 % Choose two mergeable clusters.
```

```
 mergeable=0; i=1; inds=zeros(1,2);
 while ((i<=(nclus-1))&(mergeable==0))
  j=i+1;
  while ((j<=nclus)&(mergeable==0))
   set1=clus{i}; n1=length(set1);
   set2=clus{j}; n2=length(set2);

   %n1=length(set1); n2=length(set2);

   % Compute the intra-cluster mean score.
   tmpW1=W(set1,set1); tmpW1=triu(tmpW1);
   tmpW2=-1*ones(length(set1),length(set1)); tmpW2=tril(tmpW2);
   tmpW=tmpW1+tmpW2;
   for k=1:length(set1)
    tmpW(k,k)=tmpW1(k,k);
   end
   v1=sum(sum(tmpW(find(tmpW>=0))));
   tmpW1=W(set2,set2); tmpW1=triu(tmpW1);
   tmpW2=-1*ones(length(set2),length(set2)); tmpW2=tril(tmpW2);
   tmpW=tmpW1+tmpW2;
   for k=1:length(set2)
    tmpW(k,k)=tmpW1(k,k);
   end
   v2=sum(sum(tmpW(find(tmpW>=0))));
   val1=(v1+v2)/((n1*(n1+1)/2)+(n2*(n2+1)/2));

   % Compute the inter-cluster mean score.
   tmpW=W(set1,set2); v3=sum(sum(tmpW));
   val2=v3/(n1*n2);

   % Compute the joint mean score.
   val3=(v1+v2+v3)/((n1*(n1+1)/2)+(n2*(n2+1)/2)+n1*n2);

   % Count the fraction of inter-cluster entries with scores >= threshold.
   f=sum(sum(W(set1,set2)>=wthre2))/(n1*n2);


   % Check if the difference of the mean correlation coefficients is
small.
   % Do not merge singletons.
   % Also do not merge if the joint cluster mean correlation <= wthre1.
   %if (((val1-val2)<=diffthre)&(n1>1)&(n2>1)&(val3>=wthre1))
   %if (val2>=0.65)
   %if (f>=0.9)
   %if (val2>=wthre2)
   if (f>=0.7)
   %if (f>=0.9)
    mergeable=1; inds=[i j];
   end

   j=j+1;
  end
  i=i+1;
 end
```

```
 % Merge two clusters.
 if (mergeable==1)

  i=inds(1); j=inds(2);
  clus{i}=union(clus{i},clus{j});
  for k=j:(nclus-1)
   clus{k}=clus{k+1};
  end
  nclus=nclus-1;
 else
  flag=0;
 end

end



%%%%%%%%%%%%%%%%%%%%%%%%%%%%%%%%%%%%%%%%%%%%%%%%%%%%%%%%%%%%%%%%%%%%%%%%%%
% find_conn_comps.m.
%%%%%%%%%%%%%%%%%%%%%%%%%%%%%%%%%%%%%%%%%%%%%%%%%%%%%%%%%%%%%%%%%%%%%%%%%%

% Find all connected components of a graph.

function [nconncomps, conncomps] = find_conn_comps(nnodes, G)

% Recursively label nodes in the graph until all nodes are labeled.
labeled=zeros(1,nnodes); flag=1;

% Label all the singletons.
k=0;
for i=1:nnodes
tmp=find(G(i,:)>0);
if (length(tmp)==0)
k=k+1; labeled(i)=k;
end
end


while (flag==1)

% Check if all nodes are labeled.
n=0;
for i=1:nnodes
if (labeled(i)==0)
n=n+1;
end
end

% If yes, then return.
if (n==0)
flag=0;

% Otherwise pick up an unlabeled node and recursively label its neighbors.
```

```
else
i=1; j=0;
while ((i<=nnodes)&(j==0))
if (labeled(i)==0)
j=i;
end
i=i+1;
end
k=k+1;
labeled=recurse_label2(j,nnodes,G,labeled,k);
end

end


% Find the connected components from the labels.
nconncomps=k; clear conncomps;
for i=1:nconncomps
conncomps{i}.n=0;
conncomps{i}.comps=[];
end

for i=1:nnodes
j=labeled(i);
conncomps{j}.n=conncomps{j}.n+1;
conncomps{j}.comps(conncomps{j}.n)=i;
end

% Sort the connected components by size.
tmp=zeros(1,nconncomps);
for i=1:nconncomps
tmp(i)=conncomps{i}.n;
end
[Y,I]=sort(tmp,'descend'); clear tmp2;
for i=1:nconncomps
tmp2{i}=conncomps{I(i)};
end
conncomps=tmp2;

% Discard the connected components which do not have type 1 or type 2
nodes.
discard=zeros(1,nconncomps);
for i=1:nconncomps
if (conncomps{i}.n<=0)
discard(i)=1;
end
end
tmp=find(discard==0);
for i=1:length(tmp)
tmp2{i}=conncomps{tmp(i)};
end
nconncomps=length(tmp); conncomps=tmp2;

clear tmp tmp2 Y I discard;
```

```
%%%%%%%%%%%%%%%%%%%%%%%%%%%%%%%%%%%%%%%%%%%%%%%%%%%%%%%%%%%%%%%%%%%%%%%%%%%
% recurse_label2.m.
%%%%%%%%%%%%%%%%%%%%%%%%%%%%%%%%%%%%%%%%%%%%%%%%%%%%%%%%%%%%%%%%%%%%%%%%%%%

% Recursively label nodes in a graph.
% Difference from recurse_label.m: not on a bipartite graph.

function labeled = recurse_label2(ind, nnodes, G, labeled, label)

% Label the target node.
labeled(ind)=label;

% Recursively visit neighbors.
tmp=find(G(ind,:)>0);

% If all neighbors are labeled, then return.
if (sum(labeled(tmp)==0)>0)
for i=1:length(tmp)
if (labeled(tmp(i))==0)
labeled=recurse_label2(tmp(i),nnodes,G,labeled,label);
end
end
end
```
